# Inferring upstream regulatory genes of FOXP3 in human regulatory T cells from time-series transcriptomic data

**DOI:** 10.1038/s41540-024-00387-9

**Published:** 2024-05-29

**Authors:** Stefano Magni, Rucha Sawlekar, Christophe M. Capelle, Vera Tslaf, Alexandre Baron, Ni Zeng, Laurent Mombaerts, Zuogong Yue, Ye Yuan, Feng Q. Hefeng, Jorge Gonçalves

**Affiliations:** 1 Luxembourg Centre for Systems Biomedicine, University of Luxembourg, Belvaux, Luxembourg; 2 Department of Infection and Immunity, Luxembourg Institute of Health, Esch-Sur-Alzette, Luxembourg; 3 Faculty of Science, Technology and Medicine, University of Luxembourg, Esch-sur-Alzette, Luxembourg; 4 Transversal Translational Medicine, Luxembourg Institute of Health, Strassen, Luxembourg; 5 School of Artificial Intelligence and Automation, Huazhong University of Science and Technology, Wuhan, China; 6 Department of Plant Sciences, University of Cambridge, Cambridge, United Kingdom; 7 Present Address: Robotics and Artificial Intelligence, Department of Computer Science, Electrical and Space Engineering, Luleå University of Technology, Luleå, Sweden

**Keywords:** Systems biology, Immunology

## Abstract

The discovery of upstream regulatory genes of a gene of interest still remains challenging. Here we applied a scalable computational method to unbiasedly predict candidate regulatory genes of critical transcription factors by searching the whole genome. We illustrated our approach with a case study on the master regulator FOXP3 of human primary regulatory T cells (Tregs). While target genes of FOXP3 have been identified, its upstream regulatory machinery still remains elusive. Our methodology selected five top-ranked candidates that were tested via proof-of-concept experiments. Following knockdown, three out of five candidates showed significant effects on the mRNA expression of FOXP3 across multiple donors. This provides insights into the regulatory mechanisms modulating FOXP3 transcriptional expression in Tregs. Overall, at the genome level this represents a high level of accuracy in predicting upstream regulatory genes of key genes of interest.

## Introduction

Understanding the mechanisms behind the dynamics of gene expression is fundamental to learn how cells work. With the development of large-scale experimental approaches, identification of the target genes of a transcription factor of interest becomes relatively straightforward^1,2^. However, the discovery of upstream regulatory genes that control or modulate expression levels of a given critical gene is so far realized in biological laboratories mainly by trial and error, which is very demanding in terms of time and resources. To this end, we applied and validated an unbiased computational approach to accelerate the discoveries of upstream regulatory genes, beyond the promoter-binding transcription factors, of a critical transcription factor in primary human cells.

In recent years, thanks to the availability of high-throughput experiments that measure the whole genome, there has been substantial progress in methods for inference of gene regulatory networks from large-scale transcriptional datasets^3–7^. In particular, crucial to infer gene networks is the accessibility of time-series data^8–12^. Those methods are usually referred to as reverse engineering methods, and they all have particular strengths and limitations^13,14^. One of the main limitations most of those methods suffer from is the lack of computational scalability and hence of the possibility to be applied at the genome-scale, rather than only on small networks. In fact, those methods usually tackle the challenging problem of inferring gene regulations within a network of N genes × N genes, which consequently constrains their applicability to a relatively small number of genes. This paper instead addresses the problem of modeling and inferring potential upstream regulatory genes of one selected gene of particular interest from all genes within the genome, i.e., the simplified problem of inferring potential N × 1 interactions. Thus, our network inference approach is able to address an unsolved and challenging question: to predict in an unbiased manner upstream regulatory candidate genes of a critical transcription factor by searching the whole genome.

Previously, we introduced a reverse engineering computational method which successfully identified causal regulatory relationships from time-series data^15–17^. That method was applied to infer small gene regulatory networks of the circadian clocks of *Arabidopsis thaliana*^16^ and Barley (*Hordeum vulgare*)^17^ and it showed similar or even better performance than several state-of-the-art methods^18^, including past DREAM challenges winners. Here, to infer potential upstream regulatory genes of a transcription factor of interest, we extended this method to consider: 1) non-oscillatory systems; 2) genome-scale transcriptional data including human datasets; and 3) delayed dynamical systems, to include indirect regulations via unmeasured species, e.g., proteins, that can delay the action of a regulatory gene. Technically, our method fits delayed first-order linear systems to limited time-series transcriptome data to unbiasedly test whether any gene regulates the particular gene under investigation. The challenge is to tune the model complexity with enough detail to establish causality and thus regulation, while at the same time to avoid overfitting, and to scale the approach to the whole genome.

The aim of this work is to showcase an original successful real-world application of a method, tailored from our previously published work^18^, to human primary T cells (see ref. ^18^ for comparisons with other state-of-the-art methods). Here, we illustrate the method on a high-frequency time-series genome-scale transcriptomic dataset^19^, to obtain candidates regulating a key transcription factor in a specific immune cell type. These data were obtained from a type of primary human immune cells known as CD4+CD25+FOXP3+ regulatory T cells (Tregs)^20,21^. Our goal is to identify potential upstream regulatory genes of the master regulator of Tregs, i.e., FOXP3. The transcription factor FOXP3 plays a decisive role for the development and function of Tregs^22^. Tregs perform immunosuppression of effector cells to induce immunological self-tolerance and maintain homeostasis^23–25^. Tregs are involved in different types of diseases, such as autoimmune diseases^26–28^, cancer^29–31^, infectious diseases^32,33^, neurodegenerative diseases^34,35^ and others^36^.

Target genes of FOXP3 have been identified through intensive studies^37–39^, together with insights into genetic and epigenetic mechanisms regulating expression or protein stability of FOXP3^40–45^. The majority of known upstream regulators of the expression of FOXP3 are general regulatory genes, e.g., those controlling interleukin signaling pathways (IL-2, IL-4, IL-6 and so on) and cell surface receptors (TGFB)^46^. Those genes tend to regulate a large number of targets far beyond FOXP3, which might cause significant unwanted off-targeting effects when being targeted.

Identifying more specific regulatory genes of the master regulator FOXP3 may be crucial for developing new immunotherapeutics against autoimmune and other related diseases. In fact, targeting master regulators of other cell types in other diseases, e.g., cancer, has demonstrated to be very promising in the development pipeline^47–49^. FOXP3 is the Treg lineage transcription factor that plays a decisive role in both Treg development and suppressor function. However, like other transcription factors, FOXP3 is located intracellularly and it is not easily targetable by classical methods. Hence, it is important to identify upstream genes of FOXP3 to see whether one could alternatively modulate the expression of FOXP3 via its upstream regulatory genes. Of particular interest is to identify extracellular candidate regulatory genes as more druggable hits for promising treatment developments.

The goal of identifying novel upstream regulatory genes of FOXP3 is challenging. A recent study on Tregs from human single-cell RNA-seq data^50^ could only identify potential genes co-expressed with FOXP3, but not its upstream regulatory genes. In fact, with data very limited in the number of time points it can be hard to establish causality, thus often simpler alternatives are employed, such as correlation, mutual information or other basic statistics^6,51^. These methods mainly identify co-expression of genes, rather than causal interactions^52^, i.e., regulations. Also, mathematical models of Tregs’ dynamics or wider parts of the immune system are not useful in finding novel regulatory genes of FOXP3 because they mainly included known regulations^53–60^. Thus, novel upstream genes regulating FOXP3 are still largely unknown. A very recent study^61^ addressed this problem experimentally, screening around five hundred predefined nuclear factors by CRISPR to identify gene regulatory programs promoting or disrupting FOXP3 expression. However, unbiased experimental screening of the whole genome for such upstream regulatory genes in a rare-frequency primary immune subset, i.e., Tregs, is still impossible even with state-of-the-art experimental approaches^62^ and available resources. Instead, such a screening task could be first performed computationally to facilitate the candidate short-list selection for followup experimental investigations. This is the goal of this work.

In summary, this paper shows that a simple dynamical model can efficiently (genome level) and unbiasedly predict upstream regulatory genes of specific targets. It suggested five genes that potentially regulate FOXP3 expression, three of which were subsequently experimentally validated. At the genome level and in primary human cells, where high heterogeneity exists among different individuals relative to the scenario in murine cells, our method exhibits a high level of predictive accuracy.

## Results

The unbiased computational screening approach used in this work is based on a method rooted in the engineering field of systems identification of dynamical systems. Here, we illustrate the use of this tool to predict regulatory genes of the Treg master regulator FOXP3 from the whole genome. The method can be applied to various types of time-series data from different organisms, including transcriptomic, proteomic and metabolomic data and others. Ideally, the time-series input data should have constant time intervals between measurements. Otherwise, data can be, for example, interpolated.

This paper used published time-series microarray transcriptomic measurements from isolated primary human Tregs^19^. The datasets consist of Tregs from two different healthy donors that were stimulated at time zero with anti-CD3/-CD28/IL-2, and measurements were taken at time zero followed by sampling every twenty minutes over a period of six hours (19 time points in total). The transcript expression was then analyzed in Affymetrix HG-U133 plus 2.0 oligonucleotide arrays (see ref. ^19^ for details). Although the number of 19 time points is already quite large and rare in biological experimental settings, this number is still limited from our engineering-based dynamical modeling perspective.

**Fig. 1:**
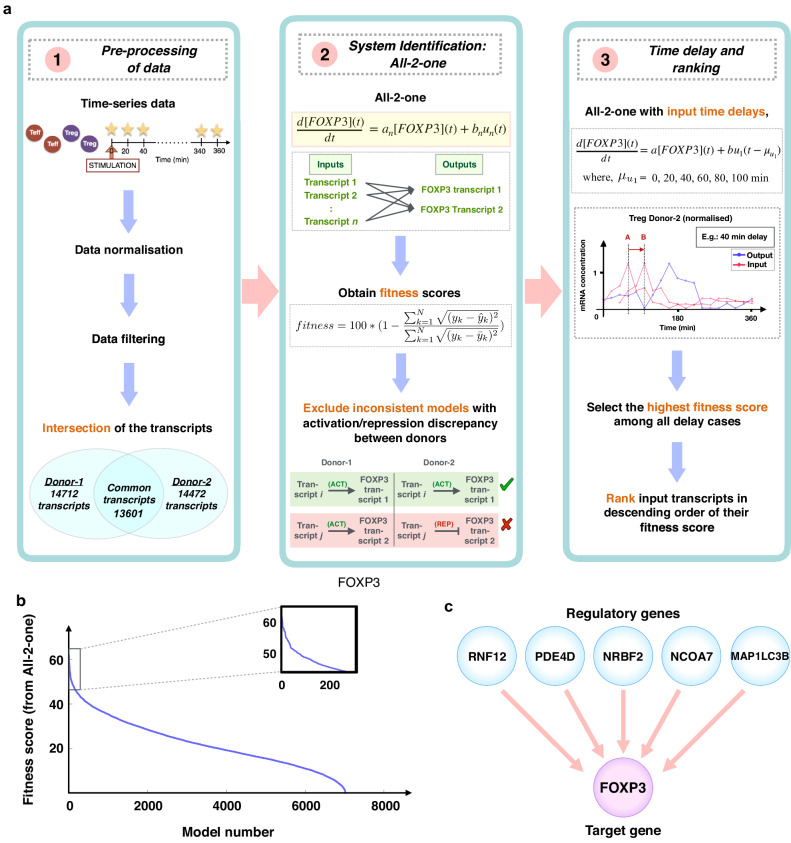
Causal dynamical modeling pipeline resulting in hypothesized upstream regulatory genes of FOXP3 for subsequent experimental testing. **a** Overview of the different stages of the computational approach, from raw time-series transcriptomic data to the resultant top ranking of genes likely to regulate FOXP3 expression in primary human Tregs. The abbreviation ACT stands for activator, REP for repressor. In the third column, the red signal A and B represents the original input signal and a delayed version, respectively. **b** The number of models that reach the given fitness score or higher. Two regimes are visible: a group of models showing a steep decrease in fitness scores, and a much larger group showing an almost linear decrease in fitness scores. The zooming-in view on the top right corner of this panel shows a magnification of the region with the top ranked models. The list of the top-ranked 176 transcripts is reported in Table 1. **c** Overview of predicted top-ranked upstream regulatory candidate genes of FOXP3 which were tested in vitro.

After pre-processing, as outlined in column 1 of Fig. 1a and described in Methods, there were 13,601 transcripts left, corresponding to 7826 genes. A gene can correspond to multiple transcripts, each measured by a separate probeset; unless otherwise stated, from now on for simplicity we will only use the term “transcript” to replace “probeset”.

The next step is to build dynamical models that capture causality. However, there are many choices on model classes and complexity. With rich data, models can be complex, providing detailed information of mechanisms of action. In our case, with only a single time-series experiment limited to 19 time points and with limited resources for validation, we considered one of the most minimalistic classes of dynamical models: first-order linear time-invariant (LTI) systems, a well established modeling strategy in small scale systems^15–18,63,64^. This simple class avoids overfitting and introducing biases.

In this particular study, we are only interested in predicting upstream regulatory candidates of one gene, i.e., FOXP3. Hence, the models take as single inputs the time-series expression values of each of the 13,601 transcripts, and FOXP3 as the single output (target). This leads to a large number of pairwise dynamical models (one per transcript), testing whether each transcript on its own could regulate FOXP3. A model associated with a particular *transcript* is given by

1 $$\frac{d\,{{\mbox{FOXP3}}}(t)}{dt}=a\cdot {{\mbox{FOXP3}}}\,(t)+b\cdot transcript(t).$$

The left-hand side of the equation is the derivative over time (i.e., the rate of change) of the mRNA concentration of FOXP3. The first term on the right-hand side mostly corresponds to degradation; it can also capture negative or positive feedback from FOXP3 itself, in case of autoregulation. The second term describes the regulation of FOXP3 gene expression by another transcript. Given the simplicity of the model, the two parameters *a* and *b* will be adjusted to best fit the data and, hence, they are more likely to represent an abstraction rather than a physical meaning. Based on this equation, we searched for parameters *a*, *b* that best fitted the data. This procedure was repeated for all 13,601 transcripts. We excluded any transcript leading to inconsistent models between the two donors in terms of its role as activator/repressor. These steps were summarized in column 2 of Fig. 1a and further details were provided in Methods.

If a potential candidate is a transcription factor, the corresponding transcript still needs to be first translated to the encoded protein and then bind to the promoter areas of FOXP3. This results in a time delay from the activation of the transcript of the candidate regulator to the mRNA expression of FOXP3. Very often, the upstream regulatory candidate is not a transcription factor, which has to go through several intermediate molecules/proteins to be able to modulate the expression of FOXP3 and therefore requires even a longer delay. To capture the dynamics of intermediate steps and avoid significantly increasing model complexity, we therefore introduced a time delay *τ* in the input signal

2 $$\frac{d\,{{\mbox{FOXP3}}}(t)}{dt}=a\cdot {{\mbox{FOXP3}}}\,(t)+b\cdot transcript(t-\tau ).$$

Overall, the model has only a total of three parameters and lower chance of overfitting when applied to the few time points available. Moreover, this simple model can scale to the whole genome. In contrast, other more complex models such as higher dimensional systems, nonlinearities, or linear combinations of multiple inputs with penalty on complexity, have a higher risk to overfit and typically lead to combinatorial optimizations (not scalable). Nevertheless, it is possible that other existing models would also lead to useful predictions.

Fitness of each model to the data was quantified by the fitness score defined in the second equation of column 2 of Fig. 1. Higher fitness values corresponded to transcripts more likely to regulate FOXP3 (fitness ranges from 0 to 100, with 100 being perfect fit). For each model, we considered several possible delays *τ* and selected the delay leading to the highest fitness. Next, all 7030 models left at this point were ranked according to this fitness. This is illustrated in column 3 of Fig. 1a and described in Methods in detail.

Next, we decided to focus on the top ranked 2.5% genes of our list, i.e., 176 transcripts (Table 1) out of 7030. Fitness scores decreased rather steeply for the top ranked models, and then decreased much slower. While any cut-off is somewhat arbitrary, we decided to focus on genes at the top of the list, since those are more likely to regulate FOXP3 (additional details in Methods). Those 176 genes included a wide variety of cell functions and allowed an individual investigation of their biological functions and dynamics.

**Table 1:** Highest ranked 176 transcripts according to our approach

Rank^a^	Gene name^b^	Delay^c^	Fitness^d^	Rank	Gene name	Delay	Fitness	Rank	Gene name	Delay	Fitness
**1**	ZNF331	100	62.14	**60**	TMEM186	0	50.46	**119**	SRSF7	100	47.79
**2**	C16orf72	40	61.91	**61**	FARS2	40	50.43	**120**	MAPKAP1	100	47.62
**3**	ANKRD49	40	61.40	**62**	CEP57	100	50.26	**121**	KLF4	100	47.60
**4**	ZNF331	100	60.49	**63**	APC	40	50.15	**122**	ZNF559	100	47.56
**5**	EXOC3L2	0	59.49	**64**	DPH5	80	50.14	**123**	LPIN2	40	47.53
**6**	SKI	40	58.27	**65**	LINC00909	40	50.08	**124**	NPRL2	100	47.53
**7**	PDE4D	80	58.19	**66**	IRS2	100	50.07	**125**	KIAA0391	100	47.53
**8**	RNF6	40	58.09	**67**	FLJ31306	20	49.91	**126**	TIPARP	100	47.52
**9**	BBX	40	58.06	**68**	SNRK	60	49.87	**127**	ING3	80	47.37
**10**	RNF144A	40	57.28	**69**	ZNF331	100	49.84	**128**	CBX4	100	47.36
**11**	PRKAR2A	40	57.25	**70**	MRPS31	100	49.76	**129**	FOCAD	100	47.30
**12**	MEF2D	100	57.17	**71**	C11orf21	100	49.73	**130**	FAM63B	40	47.25
**13**	IRS2	100	57.12	**72**	OSBPL7	100	49.72	**131**	OSBPL2	40	47.18
**14**	CCDC109B	40	56.79	**73**	ZSCAN16	40	49.72	**132**	DFFA	100	47.17
**15**	MTMR12	40	56.73	**74**	FAM13A	100	49.71	**133**	GCFC2	80	47.12
**16**	EHBP1	40	55.84	**75**	ZNF764	40	49.59	**134**	BCL11B	100	47.11
**17**	AGGF1	40	55.19	**76**	CYP2R1	40	49.52	**135**	RLIM	100	47.09
**18**	MEF2D	100	55.05	**77**	BCL11B	100	49.41	**136**	229447_x_at	100	47.00
**19**	DUSP7	100	54.88	**78**	RNF113A	100	49.32	**137**	PCYOX1	100	46.99
**20**	SRXN1	0	54.87	**79**	COA5	100	49.31	**138**	R3HDM2	100	46.98
**21**	OTUD4	0	54.77	**80**	CITED2	100	49.22	**139**	MKKS	100	46.96
**22**	RLIM	80	54.63	**81**	RIPK2	40	49.06	**140**	HINT1	100	46.94
**23**	MTHFD1	100	54.56	**82**	EGR1	80	49.01	**141**	HERC4	100	46.84
**24**	FAM162A	100	54.32	**83**	C1orf132	100	49.01	**142**	ATPAF1	100	46.79
**25**	TIPARP	100	54.10	**84**	CD44	40	48.99	**143**	UGP2	100	46.79
**26**	PAM	100	53.98	**85**	TM2D2	100	48.97	**144**	BUD13	40	46.75
**27**	CLUAP1	100	53.93	**86**	OXNAD1	100	48.90	**145**	FAM213B	100	46.74
**28**	C11orf73	100	53.89	**87**	DERA	100	48.87	**146**	RPL34	20	46.60
**29**	PEX19	100	53.61	**88**	PPIL3	100	48.84	**147**	CD33	100	46.60
**30**	ATF3	80	53.42	**89**	SNX20	100	48.75	**148**	203359_s_at	100	46.60
**31**	PRKAR2A	40	53.29	**90**	PEX19	100	48.63	**149**	METTL5	100	46.53
**32**	DUSP10	80	53.22	**91**	ZNF331	100	48.63	**150**	217317_s_at	100	46.45
**33**	FOCAD	100	52.88	**92**	C14orf166	100	48.63	**151**	FAM13A	100	46.44
**34**	FAM213B	100	52.87	**93**	FAM162A	100	48.63	**152**	SLC35F6	0	46.42
**35**	1562056_at	0	52.69	**94**	SLC46A3	40	48.60	**153**	DBR1	40	46.39
**36**	KIAA0232	40	52.26	**95**	CD164	20	48.56	**154**	MAP1LC3B	20	46.39
**37**	CBX4	100	52.13	**96**	NRBF2	60	48.55	**155**	ACYP2	0	46.35
**38**	MRPS22	40	51.66	**97**	ZNF350	60	48.54	**156**	NUDT15	100	46.34
**39**	PHF17	40	51.64	**98**	DERA	100	48.48	**157**	PDE4D	80	46.33
**40**	SNX18	80	51.57	**99**	THEM4	100	48.47	**158**	GOLGB1	40	46.32
**41**	CGRRF1	100	51.47	**100**	ZMYM4	100	48.42	**159**	SETD7	100	46.31
**42**	ABHD13	40	51.46	**101**	RPF1	100	48.39	**160**	NSMCE2	100	46.25
**43**	GLT8D1	100	51.40	**102**	CFLAR	0	48.36	**161**	MORF4L1	80	46.16
**44**	KIAA2018	40	51.32	**103**	PCYOX1	100	48.35	**162**	CASP6	100	46.15
**45**	MPPE1	40	51.29	**104**	DUSP1	100	48.33	**163**	ATG2A	100	46.13
**46**	GGNBP2	40	51.27	**105**	ACOX1	100	48.32	**164**	RBBP6	100	46.09
**47**	MOAP1	100	51.20	**106**	ING3	80	48.29	**165**	UBFD1	100	46.05
**48**	PTGER4	40	51.14	**107**	IRF1	100	48.23	**166**	222021_x_at	100	46.05
**49**	ZNF273	80	51.13	**108**	PIK3R1	100	48.17	**167**	KLF6	80	46.04
**50**	ENO2	20	50.98	**109**	KLF6	80	48.14	**168**	GDE1	80	45.99
**51**	HERPUD1	100	50.91	**110**	NCOA7	40	48.09	**169**	KDM2A	80	45.98
**52**	IKZF4	0	50.80	**111**	BNIP3	100	48.08	**170**	FAM162A	100	45.93
**53**	CGRRF1	100	50.77	**112**	SQRDL	100	48.04	**171**	GIMAP4	100	45.92
**54**	231061_at	40	50.76	**113**	S100A10	80	48.02	**172**	NCF2	100	45.91
**55**	ARG2	100	50.72	**114**	RNF6	40	48.02	**173**	DUSP7	100	45.90
**56**	CDS2	40	50.67	**115**	RBBP6	100	48.01	**174**	MRPL39	40	45.88
**57**	LPIN2	40	50.63	**116**	ZADH2	100	47.98	**175**	COQ5	100	45.83
**58**	FAM162A	100	50.61	**117**	KIF11	100	47.97	**176**	INPP4A	100	45.78
**59**	KLF4	100	50.55	**118**	TGS1	0	47.95				

^a^Ranking in descending order based on fitness scores; 176 transcripts correspond to 161 genes.

^b^Gene symbol name or Affymetrix probe-ID (a unique identifier).

^c^Time delay (in min) in the model which received the highest fitness score among different possible time delays for the corresponding transcript as a possible upstream regulatory candidate gene of FOXP3 for the second donor.

^d^Fitness score (in percentage) as defined in Eq. (4) for that model.

Of those 176 models, there were 161 distinct genes. These genes covered fitness values ranging from 46 (lowest) to 62 (highest). Among these remaining 161 top ranked genes, FOXP3 is known to bind to the promoters of 59 genes^65^, and 38 genes are reported to be differentially expressed in human Tregs compared to CD4+CD25− effector T cells (Teffs)^65^. For 15 genes, both statements were true. As a literature validation test, this shows that the predicted top ranked genes are indeed involved in the pathways related to FOXP3 regulation, supporting the relevance of our predictions in general.

Focusing on specific examples, in the list of top ranked genes, our algorithm has predicted some candidate genes which have already been shown to play an important role in regulating FOXP3 expression or Treg suppressive function. For example, as shown in Table 1, our algorithm ranked IKZF4 as the top 52th candidate regulatory gene that could regulate FOXP3 expression. Excitingly, selective deletion of IKZF4 in Tregs leads to loss of suppressive function and development of systemic autoimmunity^66^. ARG2 was ranked 55th. It has been shown that ARG2 enhances Treg suppressive capacity in vitro and confers a selective advantage for accumulation in inflamed tissues in vivo^67^. CD44 was ranked 84th in our list. Tregs from CD44-knockout mice demonstrate impaired regulatory function ex vivo and depressed production of IL-10 and cell-surface TGF-beta^68^. IRF1 was predicted by our algorithm as one of the top ranked genes (107th). It has been reported that IRF1 negatively regulates Treg differentiation by repressing FOXP3 expression^69^.

The next step was to experimentally validate some of the 161 top ranked genes. Due to capacity constraints, it was only possible to experimentally validate five of them. Since the fitness scores of those top ranked 161 genes were relatively close, we performed the selection also based on the following additional considerations. First, probesets corresponding to more than one gene were excluded, since it is not possible to distinguish which genes produce the resulting expression profile (i.e., cannot determine exact candidates). Second, we excluded noisy time-series signals and focused only on relatively smooth time-series signals, with clearly visible rise and fall dynamics ahead of FOXP3. Third, we gave preference to transcripts with optimal delays *τ* in Eq. (2) between 20 and 60 min, considering the typical amount of time taken to translate mRNA of the regulatory transcript into protein, bind to the promoter of FOXP3 and consequentially regulate its expression. Fourth, we performed a final selection based on potential biological relevance. We gave preference to transcription regulatory genes with encoded proteins located in the nucleus, since the known regulatory pathways of FOXP3 are predominantly occurring there (see e.g., ref. ^46^).

Following these additional considerations, the top five previously-unrecognized candidates selected for experimental validation were: *NCOA7*, *NRBF2*, *PDE4D*, *MAP1LC3B* and *RNF12* (also known as *RLIM*) (Fig. 1c). The first three encoded proteins located in the nucleus. More details on this selection are provided in Methods.

**Fig. 2:**
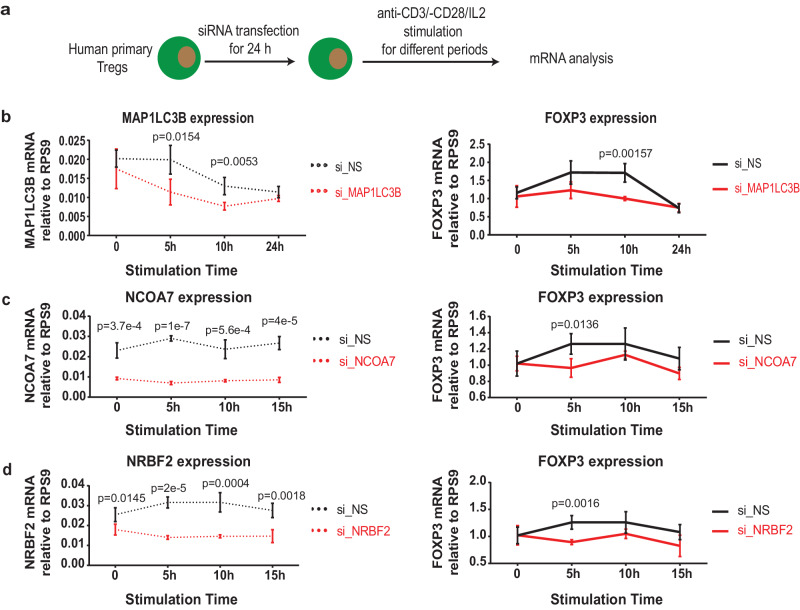
Experimental validation of predicted upstream regulatory candidate genes. **a** Schematic of the proof-of-concept validation experiments. The predicted candidate genes were knocked-down by siRNA transfection in primary human Tregs for 24 h, followed by anti-CD3/-CD28/recombinant human IL-2 stimulation for different periods. **b** Quantitative real-time PCR (qPCR) results for the knockdown of MAP1LC3B in human primary Tregs and the corresponding FOXP3 expression. Control scrambled non-specific knockdown (si_NS) is shown in black and the specific knockdown (si_MAP1LC3B) in red. **c** qPCR results for the knockdown of NCOA7 and the corresponding FOXP3 expression. Control knockdown (si_NS) is shown in black and the specific knockdown (si_NCOA7) in red. **d** qPCR results for the knockdown of NRBF2 and the corresponding FOXP3 expression. Control knockdown (si_NS) is shown in black and the specific knockdown (si_NRBF2) in red. Of note, the NCOA7 and NRBF2 knockdown experiments were performed together with the same control samples for the same donor. Statistical significance (*P*-value) was determined using two-tailed unpaired Student’s *t* test without multiple comparison correction. Unlabeled, non-significant. Displayed data are mean ± standard deviation (s.d.) for four technical replicates. Experiments have been independently repeated in 8 adult healthy donors.

Our experimental results showed a successful knockdown of *MAP1LC3B*, *NCOA7* and *NRBF2* using siRNA specifically targeted against the corresponding gene relative to a control scrambled siRNA in sorted primary human Tregs (refer to Methods, Supplementary Fig. 1 and Fig. 2a–d). The potential off-target risk for the used siRNA was minimized through the implementation of the Qiagen HP onGuard siRNA Design pipeline. Furthermore, no universal effects were observed for the employed siRNA against any of these five candidate regulatory genes on mRNA levels of an irrelevant gene, e.g., *CD4*, in Tregs (Supplementary Fig. 2). We also did not observe a significant effect on cell viability following transfection of those specific siRNAs in our project (as exampled in Supplementary Fig. 5a). As predicted by our method, partial knockdown of *MAP1LC3B*, *NCOA7* and *NRBF2* already significantly down-regulated the transcript expression of *FOXP3* in Tregs (Fig. 2b–d). The dynamics of *FOXP3* expression following siRNA treatment was slightly different for the three candidates. For the other two candidates, *PDE4D* and *RNF12*, although we successfully knocked down their mRNA expression, there was no clear effect on the mRNA expression of FOXP3 (data not shown here).

Next, we tested the potential effect on protein levels of FOXP3, although we made no model or prediction for protein levels (all models were built from mRNA data only, not to predict protein expression). We used two different protein analysis techniques, immunoblotting and flow cytometry (Supplementary Figs. 3–5). Due to the limited availability of reliable antibodies of MAP1LC3B, we performed Western blotting (WB) analysis to test the effects of knockdown of only NCOA7 and NRBF2. We observed a downregulation of NRBF2 protein levels following NRBF2 siRNA knockdown (also refer to Supplementary Note 1), which resulted in expression reduction of certain FOXP3 isoforms using WB (Supplementary Fig. 3). Since WB can only measure averaged results in bulk protein samples, we also employed single-cell based flow cytometry. In other donors, however, flow cytometry analysis exhibited no reduction in FOXP3 protein expression among gated living cells, although the same donors still showed a significant reduction on FOXP3 transcripts in Tregs with NRBF2 knockdown at 5h following stimulation as quantified by quantitative real-time PCR (qPCR) (Supplementary Figs. 4 and 5). Nevertheless, Tregs were successfully stimulated as the protein expression of another key Treg gene CTLA4 was indeed increased following stimulation in both groups (Supplementary Fig. 5c). In summary, we were unable to demonstrate whether NRBF2 could also regulate FOXP3 protein expression or not due to the results inconsistency between the two protein analysis methods. It is possible that complicated post-transcriptional and post-translational regulations explain the observed difference between mRNA and protein results.

These experiments have been repeated in Tregs isolated from peripheral blood on 8 adult healthy donors. The number of analyzed donors varied depending at which level (protein or mRNA or both) the validation was performed. The effect was not observed in up to two of the tested donors, possibly due to the heterogeneous nature of human individuals.

## Discussion

The purpose of this work is to demonstrate that applying a systems identification-engineering based computational method can unbiasedly and effectively predict upstream regulatory candidates of a gene of interest. The predictive accuracy was presented with the analysis of the top ranked candidate genes through proof-of-concept experiments. We illustrated the method with human Treg transcriptomic time-series data to identify potential regulatory genes of the master regulator FOXP3 from the whole genome. Then, we tested five selected top ranked genes via proof-of-concept experiments. Of those, three candidates were successfully validated.

Although NRBF2 knockout mice do not show spontaneous autoimmune phenotypes, NRBF2 positively regulates the autophagy process^70^, which has been widely associated with autoimmune diseases^71^. Moreover, an integrative meta-analysis from around 72 million genome-wide functional associations shows that NRBF2 is ranked as the sixth candidate gene related to juvenile rheumatoid arthritis^72^, one of the classic autoimmune diseases. NRBF2 is also known to regulate the activity of VPS34^70^. Moreover, T-cell specific depletion of VPS34 significantly impairs the maintenance and the suppressor function of Tregs^73^. Our results, together with these published works, indicate that NRBF2 might be a promising upstream regulatory gene that modulates the expression of FOXP3. To understand the physiological and pathological effects of NRBF2 on Tregs in vivo requires further investigation using NRBF2 whole-body or Treg-specific knockout mice. However, under homeostatic conditions, NRBF2-deficient mice do not develop spontaneous autoimmune phenotypes^70^. Hence, additional efforts are required to investigate the effects under induced inflammatory, infection or autoimmune disease animal models and/or during the aging process^74^ in future experiments. If successful, our work will pave the way for identifying new potential regulatory candidates to modulate the expression of FOXP3 in Tregs and various complex diseases, in which Tregs are heavily involved.

The models in this paper were fitted to transcriptional data. Hence, the models can possibly only make reliable predictions at the mRNA level. Not surprisingly, we observed that protein levels do not always follow the same dynamics as mRNA due to tight post-transcriptional and post-translational regulations of various proteins (e.g., that of FOXP3 as reviewed elsewhere^75,76^). Furthermore, owing to the existence of possible compensatory in-parallel pathways regulating FOXP3 protein expression, the partial knockdown of NRBF2 at the mRNA level might be sufficient to downregulate FOXP3 transcript expression, but not to the extent of decreasing FOXP3 protein levels. Although the siRNA we used was based on the Qiagen HP onGuard siRNA Design pipeline that has implemented various sophisticated algorithms to minimize off-target risks, it might be still worth to test different siRNAs against the same candidate genes. Furthermore, as the three candidate regulatory genes (e.g., MAP1LC3B, NCOA7 and NRBF2) are not among known transcription factors^77,78^, it is very likely that the three candidate genes might regulate the expression of FOXP3 via intermediate factors or regulations, e.g., protein-protein interactions, rather than direct promoter-binding mechanisms. Future experimental data that also measure high-resolution time-course protein, phosphorylation, acetylation and methylation levels could be included in the modeling pipeline to help shed more light on more complex regulatory mechanisms, together with more efficient genetic perturbation approaches, e.g., CRISPR knockout systems.

From the computational perspective, one limitation is that, being based on linear dynamics, our approach might miss complex non-linear interactions. Moreover, since our method checks one transcript at a time as a potential regulatory gene of FOXP3, it might miss interactions requiring cooperativity among several transcription factors or co-activators or co-repressors at a time. Regulations with multiple regulators controlling the same target gene are indeed biologically relevant^79^. Our approach could easily be extended to consider both non-linearities and multiple regulators controlling the same target gene. Non-linear and multiple-input single-output models could be included using regularization techniques to promote sparseness. However, this would likely lead to computationally-complex inference methods, limiting the applicability of the method to a considerably smaller number of genes. Moreover, due to typically limited availability of time-series data, a higher model complexity risks overfitting and increases false positives, which we aimed to minimize. This is the reason why we preferred a low number of parameters and higher confidence models.

Further potential developments include the following: (1) to experimentally test *MAP1LC3B*, *NCOA7* and *NRBF2* in vivo in animal models, for example using corresponding conventional or T-cell-specific conditional knockout mice under homeostatic and pathological conditions; (2) to experimentally investigate additional regulatory genes of FOXP3 from the predicted list of top ranked genes; (3) to apply the same computational approach to identify upstream regulatory genes of other important genes (e.g., CTLA4) for Tregs; (4) to combine available protein-protein interactions and directed regulatory interactions from public or commercial databases to infer complex regulatory interactions^80–83^; and (5) to use more complex models capturing mechanistic details if new types of time-series data under different experimental conditions become available, including single-cell RNA-seq data^84,85^.

This work presented the application of our approach to perform inference of potential regulatory candidates of a target gene of interest across the whole genome from time-series data. In particular, the use of time delays allowed us to search for hidden and more complex dynamics, while keeping the method scalable with just one additional parameter. The method was illustrated to rank the whole genome for the most likely upstream regulatory genes of FOXP3 in primary human Treg cells. To support this ranking, three out of five selected candidate genes were validated via proof-of-concept experiments at the transcriptional level, showing consistency across multiple donors. Silencing any of the following three genes, *MAP1LC3B*, *NCOA7* and *NRBF2*, down-regulated the transcript expression of *FOXP3*.

Overall, our results further enhance our understanding of the upstream regulatory networks of FOXP3, with the potential to develop new immunotherapeutics to modulate the expression of the master regulator FOXP3 in Tregs using either small molecular compounds or biologics, which might have important implications in many complex diseases. Although we only tested several intracellular candidate regulatory genes, the top ranked lists indeed contained some extracellular cell-surface candidates that could be relatively easily targeted, upon various layers of successful validations. Our results are solely derived from *human* primary T cells and therefore guarantee their translational potential. Computationally, our study showcases the power of dynamical modeling to unbiasedly infer the upstream regulatory genes of transcription factors of interest in primary cells, via screening the whole genome. The applied method could facilitate the otherwise long-lasting and resource-demanding laboratory process to identify upstream regulatory genes.

## Methods

### Time-series data normalization and filtering

As shown in Fig. 1, we obtained the raw data generated from primary human Tregs as described in ref. ^19^. Therein, microarray measurements were performed for every 20 min over the period of 6 h, after stimulation by anti-CD3/-CD28/human rIL-2 at time 0 h on Tregs from two donors (here referred to as donor-1, donor-2). This data contained 54676 transcripts/probesets for each donor, mapping various transcript variants of almost each gene in the whole genome. For simplicity, we only used the term “transcript” and skip the more technical term “probeset”, keeping in mind that they are in a one-to-one relation, thus exchangable. Although RNA-seq outperforms microarray in detecting low-abundant transcripts^86^, typically these techniques are concordant in identifying differentially expressed genes or enriched pathways^87^. Since we do not focus on low-abundant transcripts, we can safely employ this microarray data set.

Before applying any system identification technique, these time-series data need to be pre-processed. This involves normalizing the data using the gcrma algorithm^88^, a standard bioinformatics tool to remove as much noise and bias as possible from the data, which is implemented in MATLAB. For this and all the other computational aspects of this work, MATLAB versions R2016a, R2016b and R2017a were used. After normalization with the gcrma algorithm, the data were transformed to 2^*x*^ (with x representing the normalized data), to get back to absolute natural values, in linear scale, which we used hereafter. The so transformed natural data were then subject to filtering. Firstly, we applied Affymetrix flag filter, where any transcript was removed if marked as *absent* in every measurement taken at each instant of time. Conversely, we kept all the transcripts for which at least one measurement taken at any instant of time was marked with *marginally present* or *present*. The second filter applied removed the transcripts for which the average intensity (of the mRNA expression, which depends on the normalization used above) is <50, or the largest intensity among measurements performed at any time is <100 (in the arbitrary units used by the gcrma algorithm). After this filtering, 14712 transcripts were left for donor-1 and 14472 transcripts for donor-2. The intersection of these two ensembles led to a common set of 13601 transcripts left.

### One-2-one method

Here, we used the methodology presented in ref. ^16^ to identify candidate regulatory genes for FOXP3. This modeling strategy uses Linear Time-Invariant (LTI) models to capture the dynamics describing the rate of change of the selected transcript with respect to another input transcript. One-2-one refers to this model that takes a single input and a single output. A linear modeling paradigm offers advantages when data are scarce. In particular, although linear models do not provide detailed structure of the whole network, they are capable of identifying regulatory interactions with a reliable degree of precision (see below). A LTI model can be generally represented by the following set of equations:

3 $$\begin{array}{l}\frac{dx}{dt}(t)=Ax(t)+Bu(t)\\ y(t)=Cx(t).\end{array}$$

The model investigates whether the rate of change of the gene expression of a particular transcript *y*(*t*) depends on the gene expression of another transcript *u*(*t*). In particular, *u*(*t*) and *y*(*t*) represented the time series of the gene expression over time of a potential regulator of FOXP3 and FOXP3, respectively. The variable *x*(*t*) represented internal dynamics (translation, transcription, etc...) that interacted with the modeled output and were required for the behaviors observed, but were not explicitly included in the model. The dimension of the vector *x*(*t*) defines the model order: in general it can be a 1-dimensional vector (direct regulation or relatively slow dynamics compared to internal dynamics), or a multi-dimensional vector (the regulation happens through intermediate steps that introduce delays and cannot be ignored).

Estimating a model involves finding *A*, *B* and *C* which produce a vector *y*(*t*) as close as possible to the real expression data. On the one hand, complex nonlinear models have the potential to capture the dynamical relationships between genes with great precision. On the other hand, high complexity can lead to overfitting (fitting noise instead of dynamics) without sufficient data or detailed knowledge such as the network topology or types of nonlinear interactions. Here, we restricted the model order to one as it was optimally estimated in^16^. Hence, *A*, *B* and *C* were scalars. Furthermore, since *y* is the measurement of a single state, *C* then consists of a scalar of value 1. System identification was performed using the function ’pem’ implemented in MATLAB to minimize the prediction error^89^.

A linear model can approximate certain nonlinear systems, often linearized around an equilibrium point. It captures how relatively small changes in inputs lead to small changes in outputs compared to the equilibrium point. However, the location of equilibrium points are unknown and depend on the gcrma algorithm removal of background noise. Hence, a constant term was added to each pairwise gene interaction modeling and estimated simultaneously with other model parameters. Technically, this was implemented as linear models with two states, where the second state is constant (i.e., it has no dynamics) and its contribution is multiplied by a single parameter. This allowed the main part of the model to focus on the most relevant dynamical information between genes, while the constant deals with the constant bias.

To remove as much as possible bias from noise, we used a standard methodology known as Prediction Error Method (PEM): determine the model parameter *θ* such that the error $e\left(t,\theta \right)=y\left(t\right)-\hat{y}\left(t| t-1;\theta \right)$ has minimal variance. In other words, PEM minimises the mismatch between the predictions made by the model with current parameters and the observed data.

Each model was characterized by a performance index that represents the capability of the model to describe the input-output relationship. To do so, we used the fitness:

4 $$fitness=100* \left(1-\frac{\sqrt{\mathop{\sum }\nolimits_{k = 1}^{N}{({y}_{k}-{\hat{y}}_{k})}^{2}}}{\sqrt{\mathop{\sum }\nolimits_{k = 1}^{N}{({y}_{k}-{\bar{y}}_{k})}^{2}}}\right)$$

where *y*_*k*_ represented the data (output), $\bar{y}$ the average value of the data, and $\hat{{y}_{k}}$ the estimated output. MATLAB function *compare* can be used to compute the fitness of the model. A fitness equal to 100% corresponds to a perfect identification. A high fitness suggests that most of the dynamics was captured.

Then, to investigate the potential regulatory genes of FOXP3, a collection of 1st order LTI models was estimated from each of the transcripts to FOXP3. In each case, the parameters were estimated so that they together provided the best possible fit to FOXP3 time course data. This step took the following form:

5 $$\begin{array}{l}\frac{d[FOXP3](t)}{dt}={a}_{1}[FOXP3](t)+{b}_{1}{u}_{1}(t)\\ \frac{d[FOXP3](t)}{dt}={a}_{2}[FOXP3](t)+{b}_{2}{u}_{2}(t)\\ ...\\ \frac{d[FOXP3](t)}{dt}={a}_{n}[FOXP3](t)+{b}_{n}{u}_{n}(t)\end{array}$$

where *n* corresponds to the amount of possible candidates. Each model was characterized by a fitness metric that ranges from 0% to 100%, representing its capability of describing the original regulatory system between genes. A gene, therefore, would be further considered as a hypothetical regulator for FOXP3 if the model obtained using one of its transcripts was capable of reproducing the expression profile of FOXP3 with a sufficient degree of precision, which should be characterized by a high goodness of fit, as defined above.

Additionally, we derived the mathematical expression of the dynamics of FOXP3 to explicitly include a delay in the modeling, such that it took the following form:

6 $$\frac{d[FOXP3](t)}{dt}=a[FOXP3](t)+b{u}_{1}(t-{\mu }_{{u}_{1}})$$

where ${\mu }_{{u}_{1}}$ is a delay chosen between 0 min and 100 min, with steps of 20 min. The choice ${\mu }_{{u}_{1}}=0$ min reduces the models to the particular case employed above.

A systematic comparison of our methodology against state-of-the-art methods with data simulating similar experimental conditions can be found in^18^.

### Applying the one-2-one to our data: all-2-one method

For the system identification we only considered the transcripts that were left after filtering, as described above. Since, the number of remaining transcripts differed between the two donors, we collected the common transcripts between the 14,712 and 14,472 transcripts (last step of column 1 of Fig. 1a). Our All-2-one algorithm used these remaining transcripts, one at a time, as an input potential regulatory candidate gene, represented by the variable ${u}_{i}\left(t\right)$ in Eq. (5)), for the system identification technique described above as One-2-one (first step of column 2 of Fig. 1a). As the output target, the two FOXP3 transcripts were used separately, one at a time. In fact, out of 3 measured transcripts of FOXP3, only two were considered here, because the third transcript associated to FOXP3 got discarded by the average intensity filter described earlier because of its very low expression. The All-2-one was repeated for each donor which gave us a total of 4 sets of All-2-one results (i.e. each input towards the same FOXP3 transcript, for each of the two donors). The results contained fitness score of each input towards each output (second step of column 2 of Fig. 1a) and an indication whether the regulatory gene was tentatively an activator or a repressor of the target gene (i.e. if increases in its expression lead to respectively increases or decreases in the expression of the target gene). This last information was derived from the sign, respectively positive or negative, of the parameter capturing this regulation, i.e. the parameter *b* in e.g. Eq. (1).

Out of the 4 sets of All-2-one results, we now combined the results of 2 FOXP3 transcripts within each donor (third step of column 2 of Fig. 1a). Then we discarded any input transcript which corresponded to two models (one in each donor) being inconsistent in showing activation or repression towards any of the FOXP3 transcripts (fourth step of column 2 of Fig. 1a). In fact, this would reflect an inconsistency between the inferred mathematical models. This yielded 3515 transcripts of inputs in each donor. Since there were two outputs, that meant 7030 models identified left (each with an associated fitness score) in each donor.

Typically, the time passing between the expression of a regulatory gene and that of its target gene is around 20–60 min. The One-2-one method tended to attribute higher fitness to models where the output signal follows the input signal pattern with one time-point delay, which in our case corresponded to 20 min. Thus, in order to identify regulations occurring longer than 20 min, we needed to modify the One-2-one method by introducing time delays in the input signals only, Eq. (6). Thus, we ran a new round of All-2-one, with delays of 0, 20, 40, 60, 80 and 100 min, i.e. equivalent to moving each input signal to the right by 20–100 min (first step of column 3 of Fig. 1a). Here, for each input-output combination (i.e. model), only the highest fitness score among all 6 possible delay cases was retained (second step of column 3 of Fig. 1a). Eventually, these fitness scores were used to rank the 7030 models in a descending order (last step of column 3 of Fig. 1, see Table 1 for the high ranking part of the list of genes). This ranking was the main result of our computational method and the higher the ranking of a gene, the more likely it is to be involved in FOXP3 regulation.

### Selection of genes for wet-lab experiments

The above mentioned results corresponded to several hypothetical regulations, in particular the higher the ranking of a gene, the more likely it is to regulate FOXP3 expression. Genes, which received a high fitness score and thus were ranked high in our list, were considered as those with the high potential to be upstream transcriptional regulators of FOXP3.

Plotting the fitness score against the accumulative model number (Fig. 1b), ranked from highest to lowest fitness, we remarked that initially the fitness decreased steeply, and then the slope slowed down and the fitness decreased almost linearly. We thus focused on this initial fast drop and kept the top ranked 2.5% models out of 7030 models, namely, 176 models (Table 1) with high fitness values ranging from 62.14 to 45.78. These 176 transcripts corresponded to 161 genes, since in 15 cases two transcripts corresponded to the same gene. This represents a number small enough that still allowed an investigation of the biology and dynamics of each gene in the list, while being large enough to consider a wide variety of cell functions.

However, testing all of these 161 genes is very difficult, due to the intrinsic limitations of available resources, and in practice we could test only up to five of these regulations in vitro. We thus only selected five such promising regulatory candidate genes for further laboratory experimental validation. Since the fitness scores of these genes were very close, we needed to choose which one to test based on other criteria, knowing that any criterion to select potential regulatory genes will be somewhat arbitrary. Thus, we proceeded with the goal of selecting genes that were both likely to be upstream regulatory candidates of FOXP3 and of potential biological relevance. We selected five candidates from a variety of scores, but all within the two extremes of 62.14 and 45.78 of the top part of our ranking.

We performed this selection of five genes potentially regulating FOXP3 to be tested in vitro by means of the following additional considerations. First, transcripts were excluded when corresponding to more than one Entrez gene ID (more than one gene name due to the shared transcripts among genes) based on the HG-U133 plus 2.0 array annotation file (http://www.affymetrix.com/, version 24, 7 March 2008^19^). Further, as already mentioned, the data of donor-2 were of higher quality (less affected by noise) w.r.t. those of donor-1, thus we only considered the data for donor-2 for selection purposes. Next, we required a reasonably clean dynamics of the time series signals, and a reasonable visually assessed dynamic behavior. Furthermore, as mentioned we introduced a time delay, and retained for each transcript only the model corresponding to the time delay providing the highest fitness for each transcript. We also considered this delay information while performing our selection. We observed the time difference between the peak of the candidate regulatory transcript and the peak of FOXP3 transcripts of donor-2. The reason behind this was that usually between the peak in mRNA production for a regulating transcript, and the peak of mRNA production of the regulated transcript, there usually need to be a certain amount of time which we considered to be reasonable between about 20–60 min in consideration of the required intermediate biological processes.

Having in these ways lowered down the 161 genes to a pool of 20, we performed a final selection on the basis of biological relevance (e.g., gene ontology, based on http://amigo.geneontology.org/amigo, and protein location inside/outside the nucleus, based on https://www.uniprot.org/). On one hand, we preferred genes related to transcription and located in the nucleus, since the transcriptional regulatory process of FOXP3 is supposed to predominantly occur in the nucleus (see e.g. ref. ^46^). Using these criteria, we selected 3 genes: NRBF2, NCOA7 and RNF12. On the other hand, we also did not want to limit ourselves to these predefined hypothesized functions and subcellular locations, so for the remaining two slots we selected genes representing different functions and with encoded proteins located outside the nucleus, namely PDE4D and MAP1LC3B.

Thus based on all the criteria above we selected five potential regulatory genes for subsequent in vitro experimental tests, namely NCOA7, NRBF2, RNF12, PDE4D and MAP1LC3B.

### Regulatory T cell isolation and culture

Informed consent was obtained from healthy blood donors through the Red Cross Luxembourg and study procedures were approved with the reference number (LIH-2022-004) by the ethic committee of the Red Cross Luxembourg. Buffy coats from adult healthy donors of unknown age were provided by the Red Cross Luxembourg. For Fig. 2, Supplementary Figs. 2 and 3, isolation of human Tregs was performed using similar methods as described in ref. ^74^. The RosetteSepac Human CD4+ T cell Enrichment Cocktail (15062, Stemcell) was added to undiluted blood at a concentration of 50 μl/ml and incubated for 30 min at 4 °C. The blood was then diluted two times with the FCM buffer [Ca2+ free PBS + 2% heat-inactivated fetal bovine serum (FBS)] and the CD4+ cells were isolated by gradient centrifugation at 1200 × *g* for 20 min, using Lympoprep (07801, StemCell) and SepMateac-50 tubes (85450, Stemcell). Primary natural regulatory T cells (CD4+CD25^*high*^CD127^*low*^) were then sorted on a BD Aria III cell sorter (BD Biosciences) following the gating strategy (Supplementary Fig. 1). Before sorting, CD4+ T cells were stained for 30 min with mouse monoclonal [RPA-T4] anti-human CD4 FITC (555346, BD Biosciences) (dilution 1:20), mouse monoclonal [M-A251] anti-human CD25 APC (555434, BD Biosciences) (dilution 1:20) and mouse monoclonal [HIL-7R-M21] anti-human CD127 V450 (560823, BD Biosciences) (dilution 1:20) at 4 °C followed by two washing steps with the FCM buffer (200 × *g*, 10 min). Of note, no live/dead staining was added for sorting. The major used reagents or kits were provided in Supplementary Table 1. For experiments analyzing the effect on FOXP3 expression with flow cytometry (Supplementary Figs. 4 and 5), Treg isolation procedure was slightly different. Briefly, human peripheral blood mononuclear cells (PBMC) of three independent donors were isolated by gradient centrifugation, using SepMate-50 tubes (85450, StemCell) and Lymphoprep (07811, StemCell) according to the manufacturer’s instructions. Peripheral blood was diluted with an equal volume of the FCM buffer and centrifuged at 1200 × *g*, room temperature (RT) for 20 min in SepMate-50 tubes filled with Lymphoprep. After three washing steps at 200 × *g*, 4 °C, 10 min, 100 × 10^6^ of isolated PBMC per donor were used for CD4+ regulatory T cell isolation using CD4+ CD25+ CD127dim/- Regulatory T Cell Isolation Kit II human (130-094-775, Miltenyi Biotec) following the manufacturer’s recommendations. First, non-CD4+ and CD127^*high*^ cells were labeled. Labeled cells were magnetically retained on the LD columns attached to the MACS separator. The unlabeled effluent CD4+ cells were collected, labeled with CD25 MicroBeads II (10 μl per 10^7^ cells) and applied onto the MS column. Unlabeled flow-through non-Treg CD4+ cells were discarded and the column was immediately flushed with the FCM buffer using a plunger to collect magnetically labeled CD4+CD25+CD127dim/- Tregs.

Sorted Tregs were cultured in IMDM (21980-032, Thermo Fisher Scientific) complete medium, supplemented with 10% heat-inactivated FBS (10500-064, Thermo Fisher Scientific), 1× Penicillin+Streptomycin (15070-063, Thermo Fisher Scientific), 1× MEM non-essential amino acids (M7145, Sigma-Aldrich) and 1× *β*-mercaptoethanol (21985-023, Thermo Fisher Scientific) in a 37 °C, 7.5% CO_2_ incubator. The medium was further supplemented with 100 U/ml of recombinant human IL-2 (PZN 2238131, Novartis) for the culture of Tregs. For a maximum duration of 6 weeks, Tregs were restimulated every 7 days with irradiated Epstein Barr virus (EBV) (strain B95-8, VR-1491, ATCC) transformed B cells (EBV-B cells), at a 1:1 ratio to expand and maintain the culture. A RS2000 X-Ray Biological Irradiator was used to irradiate the EBV-B cells (Rad Source Technologies) for 30 min with a total of 90 Gy. On a regular basis, Tregs were characterized by flow cytometry for their expression of CD4, CD25, FOXP3 and Helios and discarded if the expression of FOXP3 and/or Helios was apparently decreased. During experiments involving flow cytometry analysis of FOXP3 expression (Supplementary Figs. 4 and 5), isolated Tregs were cultured as described above, except for the cell stimulation with ImmunoCult Human CD3/CD28 T Cell Activator (10971, Stemcell) soluble antibody (25 μl/ml) the next day following isolation.

### Flow cytometry for Treg characterization

Extracellular markers were stained in FACS buffer for 30 min at 4 °C, followed by three washing steps (200 × *g*, 10 min). Fixation, permeabilization and staining of intracellular markers was performed using the True-Nuclear Transcription Factor Buffer Set (424401, BioLegend) and following the manufuacturer’s instructions. The antibodies used for the characterization, as described elsewhere^74^, are the following: mouse monoclonal [RPA-T4] anti-human CD4 BUV395 (564724, BD Biosciences) (dilution 1:100), mouse monoclonal [M-A251] anti-human CD25 FITC (555431, BD Biosciences) (dilution 1:100), Hamster monoclonal [22F6] anti-human/mouse Helios Pacific blue (137220, BioLegend) (dilution 1:100) and mouse monoclonal [206D] anti-human FOXP3 Alexa Fluor 647 (often labeled as APC) (320114, BioLegend) (dilution 1:20). LIVE/DEAD® Fixable Near-IR Dead Cell Stain (L10119, Thermo Fisher Scientific) (dilution 1:500). CD127 antibody information: mouse monoclonal [A019D5] anti-human CD127 BV711 (351328, BioLegend) (dilution 1:50).

### Treg siRNA knockdown and stimulation

The detailed siRNA based knocking-down approach has already been described in our previous works^74,90^. To ease the comprehension, we described the procedures here again. The P3 Primary Cell 4D-Nucleofector X Kit L (V4XP-3024, Lonza) was used for the knockdown of the targeted genes, using 90 μl P3 Primary cell solution plus 100 pmol of corresponding si_RNA (resuspended in 10 μl RNAse-free H2O): si_Non-Specific (si_NS or si_CTRL) (sc-37007, Santa Cruz), si_NRBF2 (SI00139118, Qiagen), si_NCOA7 (SI02649668, Qiagen), si_MAP1LC3B (SI04200735, Qiagen), si_PDE4D (SI05587666, Qiagen), si_RNF12 (SI00113582, Qiagen). Of note, the same control siRNA samples were shared for the FOXP3 mRNA quantification among different gene knockdowns of the same donor. The Amaxa 4D-Nucleofector X System (Lonza) was used to perform the electroporation and siRNA transfection according to the manufacturers recommended program (E0-115) for primary human T cells. After transfection, Tregs were transferred into a 12-well plate with pre-warmed complete medium, supplemented with 100 U/ml IL-2, and kept at 37 °C for 24 h before being stimulated with 25 μl/ml of soluble antibodies (Immunocult Human CD3/CD28 T Cell Activator) (10971, Stemcell) in a 24-well plate for different periods. During the flow cytometry experiments, knockdown of the NRBF2 gene was performed as described above, using si_NS and si_NRBF2. 24 h after the knockdown, Tregs were unstimulated or stimulated for different periods. Stimulation was performed with 25 μl/ml of human CD3/CD27 T cell activator soluble antibodies (Immunocult Human CD3/CD28 T Cell Activator) (10971, Stemcell) plus IL-2.

### RNA extraction

RNA was extracted using the RNeasy Mini Kit (74106, Qiagen), following the manufacturers instructions and including the digestion of genomic DNA with DNAse I (79254, Qiagen). The cells were lysed in RLT buffer (Qiagen), supplemented with 1% beta-Mercaptoethanol (63689, Sigma-Aldrich). The NanoDrop 2000c Spectrophotometer (Thermo Fisher Scientific) was used to measure the RNA concentration and RNA quality was checked by assessing the RNA integrity number (RIN) using the RNA 6000 Nano kit (5067-1511, Agilent) in the 2100 Bioanalyzer Automated Analysis System (Agilent), according to the manufacturers protocol. Only the samples with a RIN of 8 or higher were considered for further analysis.

### cDNA synthesis

The detailed qPCR related methods have already been described in our previous works^74,90^. For an easier understanding, we described the procedures here again. A maximum of 500 ng of RNA was used for human cDNA synthesis, using the Superscipt^TM^ IV First Strand Synthesis System (18091050, Thermo Fisher Scientific) and following the manufacturers instructions. For the first step the mastermix contained following components (per sample): 0.5 μl of 50 μM Oligo(dT)20 primers (18418020, Thermo Fisher Scientific), 0.5 μl of 0.09 U/μl Random Primers (48190011, Thermo Fisher Scientific), 1 μl of 10 mM dNTP mix (18427013, Thermo Fisher Scientific) and RNAse free water for a final volume of 13 μl in 0.2 ml PCR Tube Strips (732-0098, Eppendorf). The reaction tubes were transferred into a C1000 Touch Thermal Cycler (Bio-Rad) or UNO96 HPL Thermal Cycler (VVR) and subjected to the following program: 5 min at 65 °C, followed by 2 min at 4 °C. For the second step, the reaction mix was supplemented with 40 U RNaseOUT Recombinant Ribonuclease Inhibitor (10777019, Thermo Fisher Scientific), 200 U SuperScript^TM^ IV (SSIV) Reverse Transcriptase (RT) (18090050, Thermo Fisher Scientific), a final concentration of 5 mM Dithiothreitol (DTT) (707265ML, Thermo Fisher Scientific) and 1× SSIV in a total reaction volume of 20 μl. The thermo cycler program for the second step was the following: 50 °C for 10 min, then 80 °C for 10 min and 4 °C until further usage. The obtained cDNA was then 5× diluted with nuclease-free water to a final volume of 100 μl. For the additional donors with flow cytometry analysis in NRBF2 knockdown experiments, the cDNA synthesis was based on the SuperScript^TM^ III, rather than IV. A maximum volume of 8 μl of extracted RNA per donor was used for cDNA synthesis using the SuperScript^TM^ III First-Strand Synthesis System (18080051, Thermo Fischer Scientific) following the manufacturer’s recommendations. Briefly, 8 μl of extracted RNA per donor was combined with 0.5 μl of 50 μM oligo(dT)20, 0.5 μl of 50 ng/μl random hexamer primers and 1 μl of 10 mM dNTP mix. For the first step, reaction tubes were transferred into the thermo cycler with the following program: 65 °C for 5 min, and then cooled on ice for 1 min. cDNA synthesis mix was then prepared by adding the components in the indicated order (per one reaction): 10× RT buffer 2 μl, 25 mM MgCl2 4 μl, 0.1 M DTT 2 μl, RNaseOUT™ (40 U/μL) 1 μl, SuperScript^TM^ III RT (200 U/μL) 1 μl. 10 μl of cDNA synthesis mix was added to each RNA/primer mixture, mixed and centrifuged briefly. The reaction tubes were transferred into the thermo cycler with the following program: 25 °C for 10 min, followed by 50 °C for 50 min. The reaction was terminated at 85 °C for 5 min and the tubes were further chilled on ice for further analysis.

### Quantitative real-time PCR (qPCR)

The reaction mix per sample for quantitative real-rime PCR (qPCR) contained: 5 μl of the LightCycler 480 SYBR Green I Master Mix (04707516001, Roche), 2.5 μl cDNA and 2.5 μl primers in a total reaction volume of 10 μl. The reaction was performed in a LightCycler 480 (384) RT-PCR platform [LightCycler 480 (384), Roche], using the LightCycler 480 Multiwell 384-well plates (04729749 001, Roche) and LC 480 Sealing Foil (04729757001, Roche). The program for qPCR was the following: 95 °C for 5 min; 45 cycles of (95 °C for 10 s, 55 °C for 10 s, 72 °C for 20 s); melting curve (65–97 °C). The results were analyzed with the LightCycler 480 SW 1.5 software. Gene specific primers used for qPCR: RPS9 (QT00233989, Qiagen) as a reference gene, NRBF2 (QT00061936, Qiagen), NCOA7 (QT00033922, Qiagen), FOXP3 (QT00048286, Qiagen), PDE4D (QT00019586, Qiagen) and MAP1LC3B (QT00055069, Qiagen). Graphpad Prism v10 was used for statistical significance analysis of qPCR results. *P*-value was determined using two-tailed unpaired Student’s *t* test without multiple comparison correction, assuming equal variance between two data groups of each comparison at the given time point.

### Western blotting

We performed Western blotting (WB) following the similar procedures as described previously in other works^74,90^. Proteins were separated in Novex WedgeWell 4–20% Tris-Glycine Gels (XP04202Box, Invitrogen), using the Novex Tris-Glycine SDS Running buffer (LC2675-4, Invitrogen). The proteins were transferred (dry transfer) using an iBlot2 Gel Transfer Device (IB21001, Invitrogen) and iBlot2 PVDF stacks (IB24002, Invitrogen). After the transfer, the membranes were blocked in 5% milk in PBS with 0.2% Tween20 (PBS-T) for 1 h at RT with gentle shaking before being incubated overnight at 4 °C with the primary antibodies: rabbit monoclonal [15H7L3] anti-human NRBF2 (702920, Thermo Fisher Scientific) (dilution 1:5000), rabbit polyclonal [FL-335] GAPDH (sc-25778, Santa Cruz Biotechnology) (dilution 1:200), mouse monoclonal [206D] FOXP3 (320102, Biolegend) (dilution 1:100), diluted in 5% BSA in PBS-T with 0.025% sodium azide. The next day the membrane was washed three times for 10 min before and after incubation with secondary goat anti-rabbit HRP-coupled antibodies (172-1019, Bio-Rad). The proteins were detected using the Amersham ECL Prime Western Blotting Detection Reagent (RPN2232, GE Healthcare Life Sciences) and visualized on the ECL Chemocam Imager (INTAS). If needed, the contrast and brightness of the obtained entire picture was adjusted using the Fiji ImageJ software. The intensity of bands on the gel was quantified based on Tiff images and the background signal was removed before normalization. The intensity as quantified in the identified corresponding peak area of each target band using the ImageJ function (Analyze/Gels) was first normalized to that of the loading control (GAPDH in this work). Following the first normalization to the loading control, the values were further divided by that of the first sample (i.e., unstimulated siRNA treated sample) detecting the corresponding target (e.g., NRBF2 or FOXP3 small/large isoform).

### Flow cytometry analysis in NRBF2 knockdown experiments

Extracellular markers were stained in the FCM buffer at 4 °C for 30 min in the dark with the following antibodies: mouse monoclonal (SK3) anti-human CD4 BUV496 (564651, BD Biosciences) (dilution 1:200), mouse monoclonal (M-A251) anti-human CD25 BUV395 (740290, BD Biosciences) (dilution 1:50), mouse monoclonal (A019D5) anti-human CD127 BV711 (351328, BioLegend) (dilution 1:50), Zombie NIR™ Fixable Viability Kit (423106, BioLegend) (dilution 1:500) followed by three washing steps at 300 × *g* for 5 min, 4 °C. Of note, the fluorochrome for some of the markers was different from that used for Treg sorting or characterization. For intracellular marker staining, cells were fixed and permeabilized with True-Nuclear Transcription Factor Buffer Set (424401, BioLegend) according to the manufacturer’s instructions. The intracellular markers were stained with mouse monoclonal (206D) anti-human FOXP3 Alexa Fluor 647 (often labeled as APC in the plots) (320114, BioLegend) (dilution 1:20) and mouse monoclonal (BNI3) anti-human CTLA4 PE-Cy5 (BD biosciences 555854) (dilution 1:20). Samples were analyzed with LSRFortessa Cell Analyzer. Results were further analyzed with FlowJo software (version 10.6.2).

### Supplementary information

Supplementary Information

reporting-summary

## Data Availability

The time-series microarray dataset analyzed in this study is already described and published in^19^ and is available in the Gene Expression Omnibus repository (GSE11292) at https://www.ncbi.nlm.nih.gov/geo/query/acc.cgi?acc=GSE11292. The raw unprocessed WB images for Supplementary Figure 3 are deposited in Mendeley accessible via 10.17632/7g3t3cjj7f.1.

## Code availability

The codes developed in MATLAB (versions R2016a, R2016b and R2017a) which implemented our computational approach and performed the described ranking of transcripts are available on the GitHub repository via https://github.com/StefanoMagni/ModellingRegulatorsFOXP3.
