# Supplementary material for: Inferring upstream regulatory genes of FOXP3 in human regulatory T cells from time-series transcriptomic data
Source: NPJ Syst Biol Appl. 2024 May 29;10:59. doi: 10.1038/s41540-024-00387-9 (PMC11137136; doi:10.1038/s41540-024-00387-9)
Supplement: Supplementary file 1 — Supplementary Information [file 41540_2024_387_MOESM1_ESM.pdf]

Supplementary Information

Inferring upstream regulatory genes of FOXP3 in human regulatory T cells from time-series transcriptomic data

Supplementary Note 1

Due to the limited availability of reliable antibodies on MAP1LC3B, we performed WB analysis to test the effects of knockdown of only NCOA7 and NRBF2 on the protein expression of FOXP3. We could successfully demonstrate the downregulation of NRBF2 protein expression. Notably, in line with the mRNA results, downregulation of NRBF2 protein expression indeed reduced the expression of certain isoforms of the FOXP3 protein (Supplementary Figure 3), possibly due to altered alternative splicing (Allan S.E. et al., 2005). As quantified in the WB gel (Supplementary Figure 3), the protein upregulation of NRBF2 that was observed in the control Treg samples in the first 24h following stimulation was flattened in the Tregs transfected with si_NRBF2.

The most clear downregulation (a 3-fold decrease) of NRBF2 protein expression in the si_NRBF2 treated samples vs. control samples was observed at 24h. Consistent with findings in (Allan S.E. et al., 2005), we observed indeed two isoforms of FOXP3 protein. Following stimulation, the bigger isoform of FOXP3 (referring to the upper band) was dramatically upregulated in the control samples with the highest normalized intensity value of 5.8, whereas that in the si_NRBF2 treated samples was kept relatively flat only with the highest value of 1.4. Among different time points, the highest diminished change fold of the big isoform of FOXP3 in the si_NRBF2 treated samples vs. the control samples was more than 6 at 24h. Even for the small isoform of FOXP3 protein (the lower band), the normalized signal intensity also showed a 6-fold reduction in the treated samples vs. the control samples at 24h, although the general signal intensity of the small isoform was much lower relative to that of the big isoform of FOXP3 following stimulation.

Primary human Treg sorting gating strategy

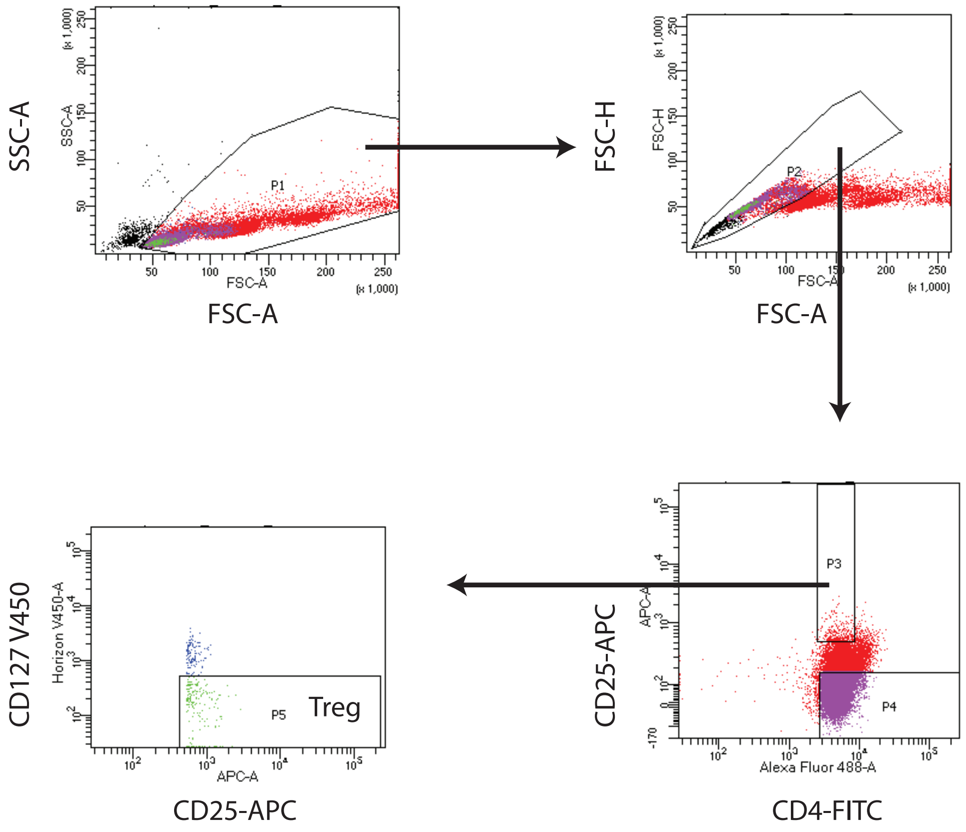

Supplementary Figure 1: **Human Treg sorting gating strategy.** Primary human Treg gating strategy. Before sorting, CD4 T cells were first enriched from PBMC. Arrow indicates the gating workflow.

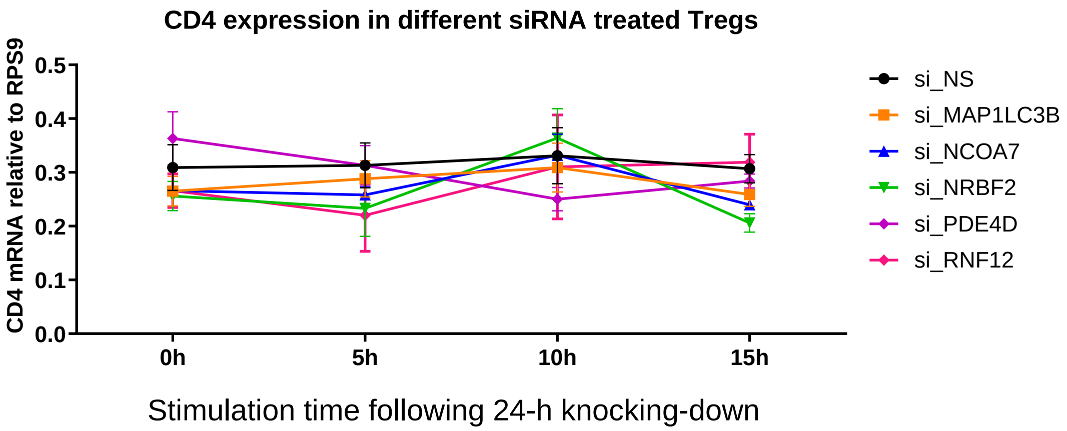

Supplementary Figure 2: **No general effect of the used siRNA on CD4 mRNA expression in Tregs. (a)** Quantitative real-time PCR (qPCR) data of CD4 mRNA for the knockdown of MAP1LC3B, NCOA7, NRBF2, PDE4D and RNF12 in human primary Tregs of one donor. Control scrambled non-specific knockdown (si_NS) is also shown. Displayed data are mean ± standard deviation (s.d.). Four technical replicates were used for qPCR results of one donor. Each experiment has been independently repeated in 7 adult healthy donors.

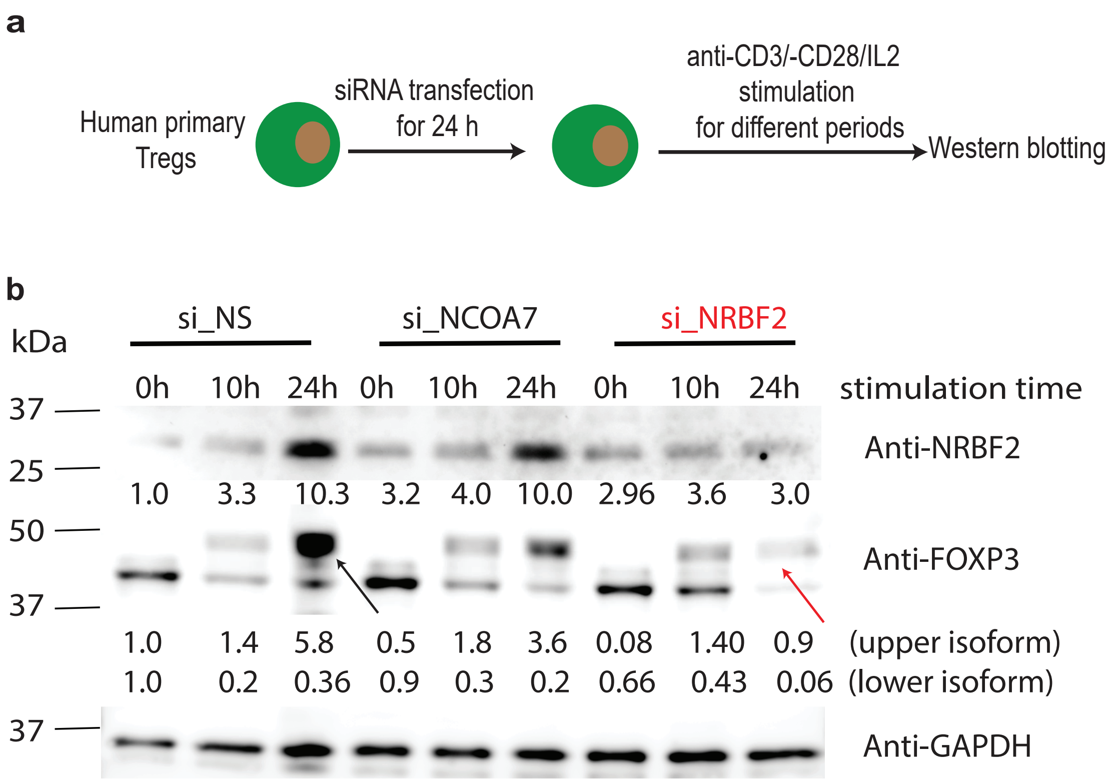

Supplementary Figure 3: **Experimental investigation of the effect of silencing candidate regulatory genes on the expression of FOXP3 proteins by Western blotting.** **(a)** Schematic of the indicated experiments. The predicted candidate genes were knocked-down by siRNA transfection in primary human Tregs for 24h, followed by anti-CD3/-CD28/human recombinant IL-2 stimulation for different periods. **(b)** Western blotting (WB) showing the protein expression of NRBF2 and FOXP3 in human primary Tregs transfected with si_NCOA7, si_NRBF2 or control siRNA (si_NS) of one donor. The bands of interest are highlighted by black (si_NS) or red arrow (si_NRBF2). The numbers below the lanes indicate the normalized intensity values relative to the loading control GAPDH. For simplicity, all the normalized intensity values in the other samples were then further normalized to that of the first sample (treated as 1). The upper and lower bands of FOXP3 protein correspond to the large and small isoforms, respectively. Molecular weights are indicated on the left of the gel. All the samples were derived from the same experiment of one donor, loaded into the same gel and processed in parallel. Each experiment has been independently repeated in 6 adult healthy donors.

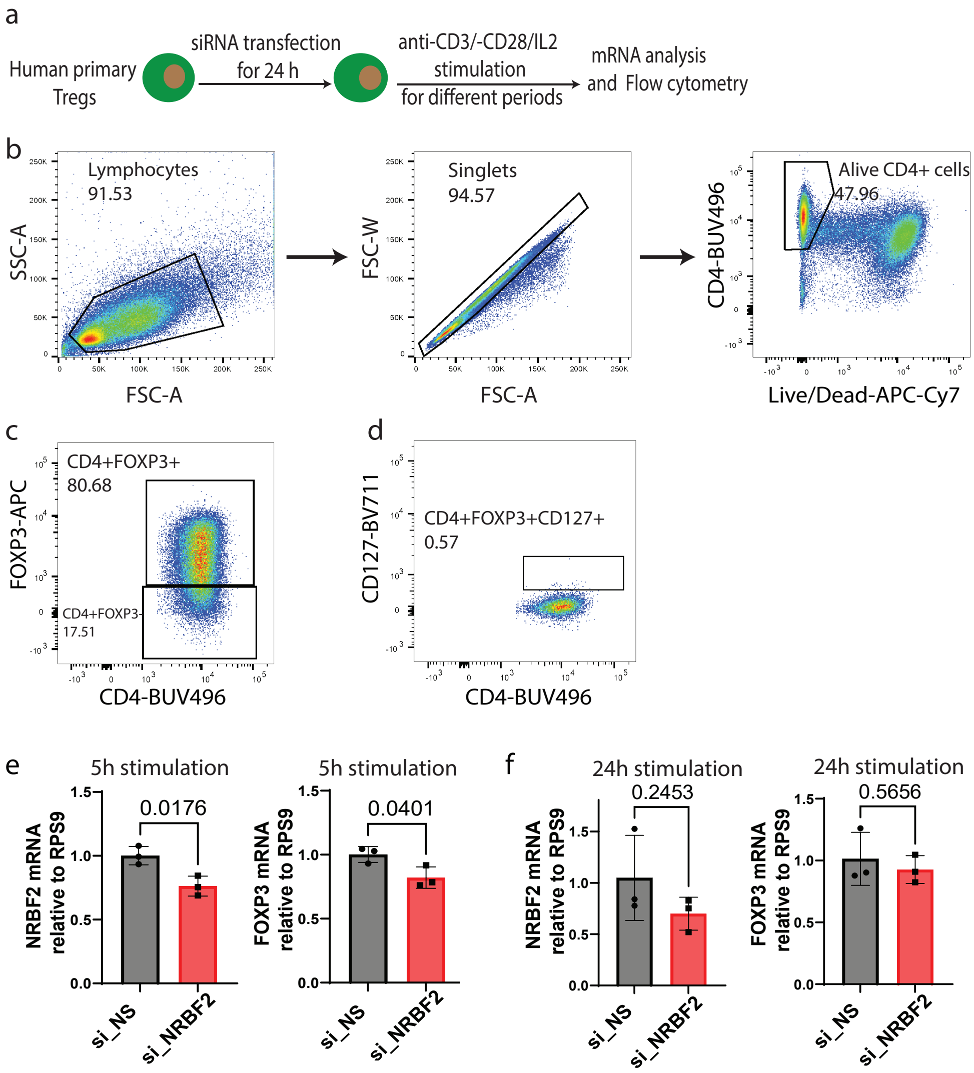

Supplementary Figure 4: **Flow cytometry characterization of isolated human Tregs and siRNA knockdown of NRBF2 in isolated human Tregs.** **(a)** Experimental procedure of the NRBF2 knockdown experiments. Primary human Tregs underwent NRBF2 gene knockdown with siRNA for 24 hours, with a subsequent stimulation by anti-CD3/-CD28 antibodies and human recombinant IL-2 for different periods. **(b)** Representative flow cytometry plots of the gating strategy from lymphocytes to living CD4+ T singlets. (c) Flow cytometry characterization of isolated human Tregs based on FOXP3 expression among living CD4+ T singlets. (**d**) Flow cytometry characterization of isolated human Tregs based on CD127 expression among living CD4+FOXP3 + singlets. (**e**) Quantitative real-time PCR (qPCR) results of the NRBF2 knockdown and the mRNA level of FOXP3 in human Tregs at 5h post stimulation. Control knockdown (si_NS) is shown in gray and the NRBF2 gene knockdown (si_NRBF2) is shown in red. (**f**) qPCR results of the NRBF2 knockdown and the mRNA level of FOXP3 in human Tregs at 24h post stimulation. Control knockdown (si_NS) is shown in gray and the NRBF2 gene knockdown (si_NRBF2) is shown in red. P values were determined with two-tailed unpaired Student's t-test without multiple comparison correction. Displayed data in e and f are mean ± s.d. from three technical replicates of each donor. Each experiment has been independently repeated in 3 adult healthy donors.

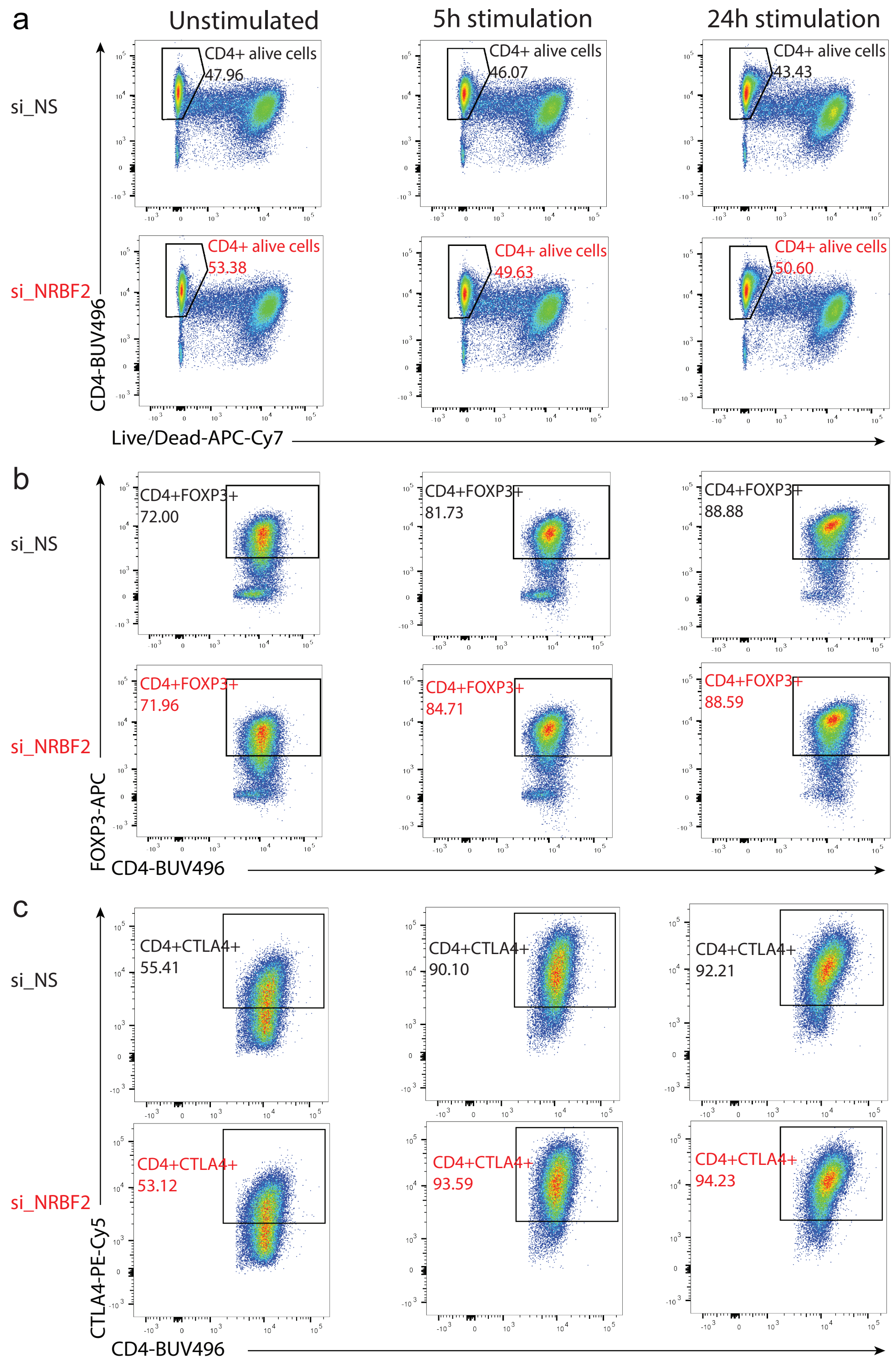

Supplementary Figure 5: **Flow cytometry analysis of FOXP3 and CTLA4 protein expression following NRBF2 gene knockdown in primary human Tregs.** **(a)** Representative flow cytometry plots of living Treg percentage at different stimulation time points with scrambled control siRNA (si_NS)- or NRBF2 (si_NRBF2)-knockdown from one donor. **(b)** Representative flow cytometry results of FOXP3 and CD4 expression in Tregs. **(c)** Representative flow cytometry results of CTLA4 and CD4 expression in Tregs. The protein expression was analyzed in unstimulated Tregs, 5h and 24h post stimulated ones with anti-CD3/-CD28 antibodies in the presence of recombinant human IL-2. Each experiment has been independently repeated in 3 adult healthy donors.

Supplementary Table 1: **List of key resources used in this work**

REAGENT or RESOURCE	SOURCE	IDENTIFIER
Antibodies (dilution factor)		
mouse mAb [RPA-T4] anti-human CD4 FITC (dilution 1:20)	BD	555346
mouse mAb [M-A251] anti-human CD25 APC (dilution 1:20)	BD	555434
mouse mAb [HIL-7R-M21] anti-human CD127 V450 (dilution 1:20)	BD	560823
mouse mAb [A019D5] anti-human CD127 BV711 (dilution 1:50)	Biolegend	351328
mouse mAb [RPA-T4] anti-human CD4 BUV395 (dilution 1:100)	BD	564724
mouse mAb [SK3] anti-human CD4 BUV496 (dilution 1:200)	BD	564651
mouse mAb [M-A251] anti-human CD25 FITC (dilution 1:100)	BD	555431
Hamster mAb [22F6] anti-human Helios Pacific blue (dilution 1:100)	BioLegend	137220
mouse mAb [206D] anti-human FOXP3 Alexa Fluor 647 (dilution 1:20)	BioLegend	320114
LIVE/DEAD Fixable Near-IR Dead Cell Stain (dilution 1:500)	Thermo Fisher	L10119
Zombie NIR Fixable Viability Kit (dilution 1 :500)	Biolegend	423106
rabbit mAb [15H7L3] anti-human NRBF2 (dilution 1:5000)	Thermo Fisher	702920
Purified mAb [206D] anti-human FOXP3 (dilution 1:100)	BioLegend	320102
mouse mAb (BNI3) anti-human CTLA4/CD152 PE-Cy5 (dilution 1:20)	BD	555854
rabbit polyclonal [FL-335] GAPDH (dilution 1:200)	Santa Cruz	sc-25778
goat anti-rabbit HRP-coupled antibodies	Bio-Rad	172-1019
Immunocult Human CD3/CD28 T Cell Activator	StemCell	10971
Bacterial and Virus Strains		
Epstein-Barr virus, strain B95-8	ATCC	VR-1491
Chemicals, Peptides, Recombinant Proteins and Assays		
Recombinant human Interleukin-2	Novartis	PZN# 2238131
RosetteSep Human CD4+ T cell Enrichment Cocktail	StemCell	15062
SepMate-50 tubes	StemCell	85450
Lympoprep	StemCell	07801
True-Nuclear Transcription Factor Buffer Set	BioLegend	424401
P3 Primary Cell 4D-Nucleofector X Kit L	Lonza	V4XP-3024
RNeasy Mini Kit	Qiagen	74106
Superscipt IV First Strand Synthesis System	Thermo Fisher	18091050
LightCycler 480 SYBR Green I Master Mix	Roche	04707516001
Novex WedgeWell 4-20% Tris-Glycine Gels	Invitrogen	XPO4202Box
Novex Tris-Glycine SDS Running buffer	Invitrogen	LC2675-4
Amersham ECL Prime Western Blotting Detection Reagent	GE Healthcare	RPN2232
**Oligonucleotides**		
Control siRNA	Santa Cruz	sc-37007
Hs_NRBF2_3 FlexiTube siRNA	Qiagen	SI00139118
Hs_NCOA7_7 FlexiTube siRNA	Qiagen	SI02649668
Hs_MAP1LC3B_8 FlexiTube siRNA	Qiagen	SI04200735
Hs_PDE4D_9 FlexiTube siRNA	Qiagen	SI05587666
Hs_RNF12_1 FlexiTube siRNA	Qiagen	SI00113582
Hs_RPS9_1_SG QuantiTect Primer Assay	Qiagen	QT00233989
Hs_NRBF2_1_SG QuantiTect Primer Assay	Qiagen	QT00061936
Hs_NCOA7_1_SG QuantiTect Primer Assay	Qiagen	QT00033922
Hs_FOXP3_1_SG QuantiTect Primer Assay	Qiagen	QT00048286
Hs_CD4_1_SG QuantiTect Primer Assay	Qiagen	QT00005264
Hs_MAP1LC3B_1_SG QuantiTect Primer Assay	Qiagen	QT00055069
Hs_PDE4D_1_SG QuantiTect Primer Assay	Qiagen	QT00019586
Hs_RLIM_1_SG QuantiTect Primer Assay	Qiagen	QT00020524
