# Supplementary material for: Inferring upstream regulatory genes of FOXP3 in human regulatory T cells from time-series transcriptomic data
Source: NPJ Syst Biol Appl. 2024 May 29;10:59. doi: 10.1038/s41540-024-00387-9 (PMC11137136; doi:10.1038/s41540-024-00387-9)
Supplement: Supplementary file 2 — reporting-summary [file 41540_2024_387_MOESM2_ESM.pdf]

nature portfolio

Corresponding author(s): Feng Q. Hefeng & Jorge GONCALVES

Last updated by author(s): Apr 30, 2024

Reporting Summary

Nature Portfolio wishes to improve the reproducibility of the work that we publish. This form provides structure for consistency and transparency in reporting. For further information on Nature Portfolio policies, see our Editorial Policies and the Editorial Policy Checklist.

Statistics

For all statistical analyses, confirm that the following items are present in the figure legend, table legend, main text, or Methods section.

n/a	Confirmed	
☐	☒	The exact sample size (*n*) for each experimental group/condition, given as a discrete number and unit of measurement
☐	☒	A statement on whether measurements were taken from distinct samples or whether the same sample was measured repeatedly
☐	☒	The statistical test(s) used AND whether they are one- or two-sided<br>*Only common tests should be described solely by name; describe more complex techniques in the Methods section.*
☒	☐	A description of all covariates tested
☐	☒	A description of any assumptions or corrections, such as tests of normality and adjustment for multiple comparisons
☐	☒	A full description of the statistical parameters including central tendency (e.g. means) or other basic estimates (e.g. regression coefficient) AND variation (e.g. standard deviation) or associated estimates of uncertainty (e.g. confidence intervals)
☐	☒	For null hypothesis testing, the test statistic (e.g. *F*, *t*, *r*) with confidence intervals, effect sizes, degrees of freedom and *P* value noted<br>*Give P values as exact values whenever suitable.*
☒	☐	For Bayesian analysis, information on the choice of priors and Markov chain Monte Carlo settings
☒	☐	For hierarchical and complex designs, identification of the appropriate level for tests and full reporting of outcomes
☒	☐	Estimates of effect sizes (e.g. Cohen's *d*, Pearson's *r*), indicating how they were calculated

Our web collection on statistics for biologists contains articles on many of the points above.

Software and code

Policy information about availability of computer code

Data collection

Flow cytometry data were acquired using the BD FACSDiva™ Software (v9.0) in LSRFortessa Cell Analyzer; The Aria III sorting acquisition software: BD FACSDiva v8.0.1.

Data analysis

Flow cytometry data were analyzed with FlowJo software (version 10.6.2).

For WB, the gel was visualized on the ECL Chemocam Imager (INTAS). If needed, the contrast and brightness of the obtained entire picture was adjusted using the Fiji ImageJ software (1.49v, https://imagej.net/Fiji/Downloads). The intensity of bands on the gel was quantified based on Tiff images and the background signal was removed before normalization. The intensity as quantified in the identified corresponding peak area of each target band using the ImageJ function (Analyze/Gels) was first normalized to that of the loading control (GAPDH in this work). Following the first normalization to the loading control, the values were further divided by that of the first sample (i.e., unstimulated control siRNA treated sample) detecting the corresponding target (e.g., NRBF2 or FOXP3 small/large isoform).

Before applying any systems-engineering identification technique, the time-series data need to be pre-processed. This involves normalising the data using the gcrma algorithm, a standard bioinformatics tool to remove as much noise and bias as possible from the data, which is implemented in MATLAB. For this and all the other computational aspects of this work, MATLAB versions R2016a, R2016b and R2017a were used. After normalization with the gcrma algorithm, the data were transformed to 2^x (with x representing the normalized data), to get back to absolute natural values, in linear scale, which we used hereafter. The so transformed natural data were then subject to filtering. Firstly, we applied Affymetrix flag filter, where any transcript was removed if marked as absent in every measurement taken at each instant of time. Conversely, we kept all the transcripts for which at least one measurement taken at any instant of time was marked with marginally present or present. The second filter applied removed the transcripts for which the average intensity (of the mRNA expression, which depends on the normalisation used above) is 50, or the largest intensity among measurements performed at any time is 100 (in the arbitrary units used by the

gcrma algorithm). After this filtering, 14712 transcripts were left for donor-1 and 14472 transcripts for donor-2. The intersection of these two ensembles led to a common set of 13601 transcripts remaining.

The detailed systems identification computational method was described in Results and Methods. The precise codes developed in MATLAB (versions R2016a, R2016b and R2017a) which implement our computational approach and perform the described ranking of transcripts are available on the GitHub repository accessible at https://github.com/StefanoMagni/ModellingRegulatorsFOXP3.

qPCR data was analyzed with the LightCycler 480 SW 1.5 software and further processed and visualized with GraphPad Prism v10.Statistical significance of PCR results was determined using two-side Student t-test without multiple comparison correction.

For manuscripts utilizing custom algorithms or software that are central to the research but not yet described in published literature, software must be made available to editors and reviewers. We strongly encourage code deposition in a community repository (e.g. GitHub). See the Nature Portfolio guidelines for submitting code & software for further information.

Data

Policy information about availability of data

All manuscripts must include a data availability statement. This statement should provide the following information, where applicable:

- Accession codes, unique identifiers, or web links for publicly available datasets
- A description of any restrictions on data availability
- For clinical datasets or third party data, please ensure that the statement adheres to our policy

The time-series microarray data set analysed in this study have already described and published elsewhere and is available in the Gene Expression Omnibus repository (GSE11292) at https://www.ncbi.nlm.nih.gov/geo/query/acc.cgi?acc=GSE11292. The raw unprocessed WB images for Supplementary Figure 3 are deposited in Mendeley via https://doi.org/10.17632/7g3t3cjj7f.1

Research involving human participants, their data, or biological material

Policy information about studies with human participants or human data. See also policy information about sex, gender (identity/presentation), and sexual orientation and race, ethnicity and racism.

Reporting on sex and gender	We cannot perform any sex/gender-specific analysis. We obtained blood samples from adult healthy donors of unknown age via the Red Cross Luxembourg. Although we requested samples ideally from males and the same blood group, Red Cross Luxembourg did not provide any sex information to us and therefore we cannot guarantee whether all the donors were males or not.
Reporting on race, ethnicity, or other socially relevant groupings	We do not have any specific population characteristics including race, age and socially relevant information.
Population characteristics	We got all the buffy coats from Red Cross Luxembourg. They are all adult healthy blood donors. We speculated that most of the blood donors are caucasian. We requested samples ideally from males and the same blood type group. But Red Cross Luxembourg could not guarantee and did not provide any detailed information to us. Precisely speaking, we do not have detailed population characteristics statistics.
Recruitment	We got samples through Red Cross Luxembourg. We did not recruit anyone ourselves.
Ethics oversight	Informed consent was obtained from healthy blood donors through the Red Cross Luxembourg and study procedures were approved with the reference number (LIH-2022-004) by the ethic committee of the Red Cross Luxembourg.

Note that full information on the approval of the study protocol must also be provided in the manuscript.

Field-specific reporting

Please select the one below that is the best fit for your research. If you are not sure, read the appropriate sections before making your selection.

☒ Life sciences ☐ Behavioural & social sciences ☐ Ecological, evolutionary & environmental sciences

For a reference copy of the document with all sections, see nature.com/documents/nr-reporting-summary-flat.pdf

Life sciences study design

All studies must disclose on these points even when the disclosure is negative.

Sample size	We did not estimate any sample size in advance. During the first phase of the project, we isolated human Tregs from 8 healthy controls. For the flow-cytometry based NRBF2 analysis, we only analyzed three healthy donors.
Data exclusions	no data exclusion was done.
Replication	For qPCR, we performed independent analysis in 8 healthy donors. For WB, we performed analysis in 6 healthy donors. The effect was not observed in up to two of the tested donors, possibly due to the heterogeneous nature of human individuals in general.

Randomization	Randomization is not applicable in this project. We did not allocate the donors into different subject groups. Instead, we performed the same analysis for each of all the involved blood donors in an anonymous manner.
Blinding	The operators performing sorting or Treg isolation were not blind to the sample pseudoID (the pseudoID, e.g., 'L24', was assigned for each donor in the laboratory only for technical convenience). We treated Tregs from different donors in the same way and cannot precisely predict the final outcomes of knocking-down any candidate genes. Therefore, this step should not affect our readouts despite the fact that the operators know the pseudoID.<br><br>The experimenters performing PCR analysis, WB and flow cytometry were not blind to the sample group allocation. The operators need to knockdown the specific candidate genes and therefore know exactly the gene specific knockdown or scrambled knockdown control groups. In any case, this should not affect our results, as all the RNA extraction, cDNA synthesis and qPCR preparation procedures were performed in the same way for all the sample groups. The machine LightCycler quantified mRNA levels in an unbiased manner. For WB analysis, all the samples were loaded into the same way and processed in parallel. The imaging device unbiasedly quantified the band signal. The ImageJ software also quantified the signal intensity of different samples in the same way.<br><br>For the flow cytometry analysis, the operators cannot influence any machine readouts in a sample-specific manner as all the complicated acquisition and quantification has to be done by the machine and the related software.

Reporting for specific materials, systems and methods

We require information from authors about some types of materials, experimental systems and methods used in many studies. Here, indicate whether each material, system or method listed is relevant to your study. If you are not sure if a list item applies to your research, read the appropriate section before selecting a response.

Materials & experimental systems

n/a	Involved in the study
☐	☒ Antibodies
☒	☐ Eukaryotic cell lines
☒	☐ Palaeontology and archaeology
☒	☐ Animals and other organisms
☒	☐ Clinical data
☒	☐ Dual use research of concern
☒	☐ Plants

Methods

n/a	Involved in the study
☒	☐ ChIP-seq
☐	☒ Flow cytometry
☒	☐ MRI-based neuroimaging

Antibodies

Antibodies used	All the used flow cytometry and WB antibodies (Abs) including the provider, cat number, clone, dilution factor, specific fluorochromes for flow cytometry were already included in Supplementary Table 1. We listed them here again.<br><br>Ab name, [clone], fluorochrome, (dilution factor), provider, reference/identification number<br>mouse mAb [RPA-T4] anti-human CD4 FITC, (dilution 1:20), BD, 555346<br>mouse mAb [M-A251] anti-human CD25 APC, (dilution 1:20), BD, 555434<br>mouse mAb [HIL-7R-M21] anti-human CD127 V450, (dilution 1:20), BD, 560823<br>mouse mAb [A019D5] anti-human CD127 BV711, (dilution 1:50), Biolegend, 351328<br>mouse mAb [RPA-T4] anti-human CD4 BUV395, (dilution 1:100), BD, 564724<br>mouse mAb [SK3] anti-human CD4 BUV496, (dilution 1:200), BD, 564651<br>mouse mAb [M-A251] anti-human CD25 FITC, (dilution 1:100), BD, 555431<br>Hamster mAb [22F6] anti-human Helios Pacific blue, (dilution 1:100), BioLegend, 137220<br>mouse mAb [206D] anti-human FOXP3 Alexa Fluor 647, (dilution 1:20), BioLegend, 320114<br>rabbit mAb [15H7L3] anti-human NRBF2, (dilution 1:5000), Thermo Fisher, 702920<br>Purified mAb [206D] anti-human FOXP3, (dilution 1:100), BioLegend, 320102<br>mouse mAb (BNI3) anti-human CTLA4/CD152 PE-Cy5, (dilution 1:20), BD, 555854<br>rabbit polyclonal [FL-335] GAPDH (dilution 1:200), Santa Cruz, sc-25778
Validation	Reaction species and applications of all the primary Abs used in this study have been validated by the given manufactures, as directly stated in the specific datasheet of the given abs. Furthermore, all the Abs were purchased from leading reliable manufactures (BD, Biolegend, or Thermo Fisher), the general routine validation statements for flow cytometry analysis are also available on the website of the corresponding provider. For BD, please visit: https://www.bdbiosciences.com/en-lu/products/reagents/flow-cytometry-reagents/research-reagents/quality-and-reproducibility; For Biolegend, please visit: https://www.biolegend.com/en-us/quality/quality-control.<br>For one Thermo Fisher Ab, the validation statement for WB application has already been provided here https://www.thermofisher.com/antibody/product/NRBF2-Antibody-clone-15H7L3-Recombinant-Monoclonal/702920.<br><br>For the provider Santa Cruz, we only used the anti-GAPDH Ab as the housekeeping loading control. It has been widely used by 622 different peer-reviewed publications (https://www.scbt.com/p/gapdh-antibody-fl-335). The quality/specificity should be very high.

Plants

Seed stocks

We did not analyze any plants in the current work. N.A.

Novel plant genotypes

N.A.

Authentication

N.A.

Flow Cytometry

Plots

Confirm that:

☒ The axis labels state the marker and fluorochrome used (e.g. CD4-FITC).

☒ The axis scales are clearly visible. Include numbers along axes only for bottom left plot of group (a 'group' is an analysis of identical markers).

☒ All plots are contour plots with outliers or pseudocolor plots.

☒ A numerical value for number of cells or percentage (with statistics) is provided.

Methodology

Sample preparation

The RosetteSepac Human CD4+ T cell Enrichment Cocktail (15062, Stemcell) was added to undiluted blood at a concentration of 50 µl/ml and incubated for 30 min at 4 degree. The blood was then diluted two times with FCM buffer (Ca2+ free PBS + 2% heat-inactivated FBS) and the CD4+ cells were isolated by gradient centrifugation at 1200 g for 20 min, using Lympoprep (07801, StemCell) and SepMateac-50 tubes (85450, Stemcell). Primary natural regulatory T cells (CD4+CD25highCD127low) were then sorted on a BD Aria III Flow cytometry cell sorter (BD Biosciences) following the gating strategy (Supplementary Figure 1). CD4+ T cells were stained for 30 min with mouse monoclonal [RPA-T4] anti-human CD4 FITC (555346, BD Biosciences) (dilution 1:20), mouse monoclonal [M-A251] anti-human CD25 APC (555434, BD Biosciences) (dilution 1:20) and mouse monoclonal [HIL-7R6 M21] anti-human CD127 V450 (560823, BD Biosciences) (dilution 1:20) 4 degree followed by two washing steps with FCM buffer (200 g, 10 min). The major reagents or kits were provided in Supplementary Table 1. For experiments analyzing FOXP3 expression with flow cytometry (results reported in Supplementary Figure 4 and 5), Treg isolation procedure was slightly different. Briefly, human peripheral blood mononuclear cells (PBMC) of three independent donors were isolated by gradient centrifugation, using SepMate-50 tubes (85450, StemCell) and Lymphoprep (07811, StemCell) according to the manufacturer's instructions. Peripheral blood was diluted with an equal volume of FCM buffer and centrifuged at 1200 g, room temperature (RT) for 20 min in SepMate-50 tubes filled with Lymphoprep. After three washing steps at 200 g, 4 degree, 10 min, 100x106 of isolated PBMC (peripheral blood mononuclear cells) per donor were used for CD4+T regulatory cell isolation using CD4+ CD25+ CD127dim/– Regulatory T Cell Isolation Kit II human (130-094-775, Miltenyi Biotec) following the manufacturer's recommendations. First, non-CD4+ and CD127high cells were labeled. Labeled cells were magnetically retained on the LD columns attached to the MACS separator. The unlabeled effluent CD4+ cells were collected, labeled with CD25 MicroBeads II (10µl per 107 total cells) and applied onto the MS column. Unlabeled flow-through non-Treg CD4+ cells were discarded and the column was immediately flushed with FCM buffer using a plunger to collect magnetically labeled CD4+CD25+CD127dim/- Treg cells.

Extracellular markers were stained in FACS buffer for 30 min at 4 degree, followed by three washing steps (200 g, 10 min). Fixation, permeabilization and staining of intracellular markers was performed using the True-Nuclear Transcription Factor Buffer Set (424401, BioLegend) and following the manufuacturer's instructions.

Instrument

For FACS sorting, we used BD Aria III sorter. BD LSRFortessa was used for analysis.

Software

The BD LSRFortessa cytometry acquisition software: BD FACSDiva 9.0; The Aria III sorting acquisition software: BD FACSDiva v8.0.1; The analysis software: FlowJO V10

Cell population abundance

The relative population abundance of each analyzed subset has been quantified by FlowJo following the provided gate strategies. The detailed frequency information has been provided through different Supplementary Figures. The gating strategy during human Treg sorting (Supplementary Figure 1): briefly, after CD4 cell enrichment, lymphocytes were gated based on SSC-A/FSC-A, then singlets based on FSC-H/FSC-A, then CD25high cells among CD4+ T cell population were identified, followed by gating and sorting of CD127-/low CD25high cells.

In average, the sorted highly-purified human primary Tregs is lower than 1% of total CD4 T cells in PBMC from the healthy donors we analyzed. The sorted human Tregs showed a purity >99% in a post-sorting analysis, when relative more Tregs were obtained. Due to the very limited number of precious Tregs for most donors, we did not always check the post-sorting purity for each experiment. In any case, we regularly checked and characterized Tregs using different markers to ensure Treg quality as described in Methods.

Gating strategy during Treg characterization and NRBF2 knockdown experiments (Supplementary Figures 4 and 5): first, lymphocytes were gated based on SSC-A/FSC-A, then singlets based on FSC-W/FSC-A, then living cells based on CD4+/ Live/Dead dye, and then FOXP3 and other relevant markers were checked. Of note, the starting materials for characterization are human Tregs.

Gating strategy

We have already provided the gating strategy examples in Supplementary Figure 1 and 4, 5.

☒ Tick this box to confirm that a figure exemplifying the gating strategy is provided in the Supplementary Information.
